# The parallel biosynthesis routes of hyperoside from naringenin in *Hypericum monogynum*

**DOI:** 10.1093/hr/uhad166

**Published:** 2023-08-17

**Authors:** Yingying Wang, Zhirong Cui, Qianqian Li, Shuai Zhang, Yongyi Li, Xueyan Li, Lingyi Kong, Jun Luo

**Affiliations:** Jiangsu Key Laboratory of Bioactive Natural Product Research and State Key Laboratory of Natural Medicines, Department of Traditional Chinese Pharmacy, China Pharmaceutical University, Nanjing 210009, China; Jiangsu Key Laboratory of Bioactive Natural Product Research and State Key Laboratory of Natural Medicines, Department of Traditional Chinese Pharmacy, China Pharmaceutical University, Nanjing 210009, China; Jiangsu Key Laboratory of Bioactive Natural Product Research and State Key Laboratory of Natural Medicines, Department of Traditional Chinese Pharmacy, China Pharmaceutical University, Nanjing 210009, China; Jiangsu Key Laboratory of Bioactive Natural Product Research and State Key Laboratory of Natural Medicines, Department of Traditional Chinese Pharmacy, China Pharmaceutical University, Nanjing 210009, China; Jiangsu Key Laboratory of Bioactive Natural Product Research and State Key Laboratory of Natural Medicines, Department of Traditional Chinese Pharmacy, China Pharmaceutical University, Nanjing 210009, China; Jiangsu Key Laboratory of Bioactive Natural Product Research and State Key Laboratory of Natural Medicines, Department of Traditional Chinese Pharmacy, China Pharmaceutical University, Nanjing 210009, China; Jiangsu Key Laboratory of Bioactive Natural Product Research and State Key Laboratory of Natural Medicines, Department of Traditional Chinese Pharmacy, China Pharmaceutical University, Nanjing 210009, China; Jiangsu Key Laboratory of Bioactive Natural Product Research and State Key Laboratory of Natural Medicines, Department of Traditional Chinese Pharmacy, China Pharmaceutical University, Nanjing 210009, China

## Abstract

Hyperoside is a bioactive flavonoid galactoside in both medicinal and edible plants. It plays an important physiological role in the growth of flower buds. However, the hyperoside biosynthesis pathway has not been systematically elucidated in plants, including its original source, Hypericaceae. Our group found abundant hyperoside in the flower buds of *Hypericum monogynum*, and we sequenced its transcriptome to study the biosynthetic mechanism of hyperoside. After gene screening and functional verification, four kinds of key enzymes were identified. Specifically, HmF3Hs (flavanone 3-hydroxylases) and HmFLSs (flavonol synthases) could catalyze flavanones into dihydroflavonols, as well as catalyzing dihydroflavonols into flavonols. HmFLSs could also convert flavanones into flavonols and flavones with varying efficiencies. HmF3′H (flavonoid 3′-hydroxylase) was found to act broadly on 4′-hydroxyl flavonoids to produce 3′,4′-diydroxylated flavanones, dihydroflavonols, flavonols, and flavones. HmGAT (flavonoid 3-*O*-galactosyltransferase) would transform flavonols into the corresponding 3-*O*-galactosides, including hyperoside. The parallel hyperoside biosynthesis routes were thus depicted, one of which was successfully reconstructed in *Escherichia coli* BL21(DE3) by feeding naringenin, resulting in a hyperoside yield of 25 mg/l. Overall, this research not only helped us understand the interior catalytic mechanism of hyperoside in *H. monogynum* concerning flower development and bioactivity, but also provided valuable insights into these enzyme families.

## Introduction

Hyperoside, namely quercetin 3-*O*-galactoside, is abundant in many edible and medicinal plants, such as roses, dodders, ginkgo leaves, hawthorns, and *Hypericum perforatum*, contributing greatly to their nutritional and medicinal values [1]. It plays a critical role in plant self-defense and breeding. For instance, hyperoside helps protect plants against UV-B radiation by scavenging reactive oxygen species [2], helps their adaption to drought, cold and salt stress [3–5], and enhances pollen fertility and the subsequent seed set by prolonging the full-blooming period and promoting pollen germination [6–8]. Moreover, hyperoside imparts important medicinal properties to these plants, making it a key pharmacological component in certain Chinese patent medicines, like Xinan capsules and Xinxuening tablets for treating heart disease and hypertension. Numerous studies have also demonstrated the notable anti-tumor, anti-fibrosis, anti-inflammatory, anti-depression, anti-viral and anti-diabetic activities of hyperoside [9]. These efficacies are important considerations in developing hyperoside as a health product and clinical drug.

*Hypericum monogynum*, a self-pollinated dicotyledonous plant belonging to the Hypericaceae family, is widely distributed in middle and southern China, Australia, and South Africa [10]. As a widely planted ornamental shrub nowadays, its functions are far more than that. The flowers can be made into flower tea to help relieve anxiety and improve sleep. The processed whole plant can be used to treat depression, trauma, inflammation, and microbial infection. The bioactive components in *H. monogynum* are mainly flavonoids, dianthrones, and phloroglucinol derivatives. A previous study reported the massive acquisition of hyperoside from the aerial parts of *H. monogynum* [11]. In previous research by our group on the chemical constituents of *H. monogynum* [12, 13], large amounts of hyperoside were found in its flower buds, which intrigued us to study the hyperoside biosynthesis mechanism in this organ.

Natural hyperoside biosynthesis involves the participation of PAL (phenylalanine ammonia lyase), C4H (cinnamic acid 4-hydroxylase), 4CL (*p*-coumaric acid:CoA ligase), CHS (chalcone synthase), CHI (chalcone isomerase), F3H (flavanone 3-hydroxylase), FLS (flavonol synthase), F3′H (flavonoid 3′-hydroxylase), and GAT (flavonoid 3-*O*-galactosyltransferase). Despite extensive studies of their genes in different plants [14–16], most of them have not been specifically characterized in hyperoside-original-source Hypericaceae plants [17] except for *HaCHS* [18], and neither has the hyperoside biosynthesis pathway been systematically elucidated in plants.

Herein, by combination of metabolic features, transcriptome sequencing, and enzymatic function verification, we revealed parallel hyperoside biosynthesis routes from naringenin in the flower buds of *H. monogynum*, which entailed the participation of HmF3Hs, HmFLSs, HmF3′H, and HmGAT. Phylogenetic analysis, molecular docking, and site-directed mutagenesis were conducted to further dissect the characteristics of these enzymes. Transcript levels and hyperoside metabolic levels indicated that hyperoside biosynthesis was more active at early flower bud stages for reproductive growth. The construction of a multi-gene co-expression recombinant *Escherichia coli* strain enabled hyperoside production from naringenin, simultaneously verifying a hyperoside biosynthesis pathway elucidated in our study. The low yield implied the need for this hyperoside-engineering strain to be remolded further by knocking out *yhhW*.

## Results

### mRNA levels and hyperoside content analysis in *H. monogynum* flower buds

**Figure 1:**
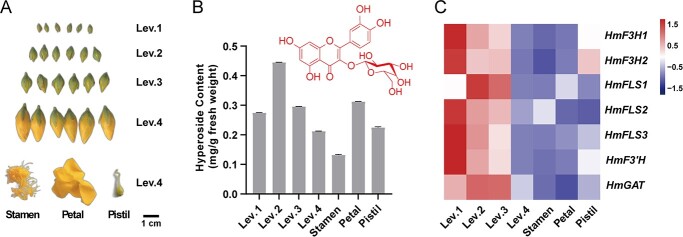
Flower buds of *H. monogynum* at different stages with corresponding metabolic and transcriptomic profiles. (A) Flower buds at different stages (Lev. 1–4) and tissues of Lev. 4 flower buds. Scale bar: 1 cm. (B) Hyperoside metabolic levels at different bud stages and tissues of Lev. 4 flower buds (without calyx). Values represent means from *n* > 3 independent samples. (C) Transcript heat map of hyperoside-biosynthesis-relevant enzymes at different bud stages and tissues of Lev. 4 flower buds (without calyx). FPKM values were normalized using the *Z*-score method.

Flower buds at different developmental stages (Level 1–4, i.e. Lev. 1-4, without calyx) and specific tissues (stamens, petals, pistils) from Lev. 4 flower buds were sampled for transcriptome sequencing and hyperoside content analysis (Fig. 1A and B). The candidate genes involved in hyperoside biosynthesis were obtained through local BLAST in the *H. monogynum* transcriptome database using reported query proteins (see Data availability section), together with gene annotations from various public databases (GO, KEGG, KOG, Nr, Nt, Pfam, Swiss-Prot) and the conserved domain analysis. Genes with relatively high FPKM (fragments per kilobase per million mapped reads) levels (>30) were chosen for further analysis. They were *HmF3H1–2*, *HmFLS1–3*, *HmF3′H*, and *HmGAT* as shown (Fig. 1C, Supplementary Data Fig. S1).

By analysis of transcription levels at different bud stages and tissues, we found that *HmF3H1–2*, *HmFLS2–3*, and *HmF3′H* had similar mRNA expression patterns (Fig. 1C, Supplementary Data Fig. S1), which were the highest at Lev. 1 stage, then decreased with the growth of flower buds. This indicated that gene expression levels were commonly active at the early stages of buds to satisfy the need for growth, fertility, and chemical defense, and were gradually downregulated when the flower buds got mature and ready to blossom. Nevertheless, *HmFLS1–2* and *HmGAT* exhibited relatively low expression levels throughout the bud stages (Supplementary Data Fig. S1). Specifically, *HmFLS1* and *HmGAT* reached their highest expression level at Lev. 2 stage, with *HmFLS1* showing more significant transcriptional activity in the petals than in the stamens or pistils of Lev. 4 buds (Fig. 1C). Corresponding to the expression pattern of *HmFLS1*, it was noteworthy that hyperoside was produced the most at Lev. 2 stage and in the petals of Lev. 4 stage (Fig. 1B), which demonstrated the direct correlation between hyperoside and HmFLS1 rather than HmFLS2–3. Therefore, HmFLS1 possibly played a primary role in hyperoside biosynthesis in *H. monogynum* flower buds.

### HmF3Hs mainly catalyze flavanones into dihydroflavonols

**Figure 2:**
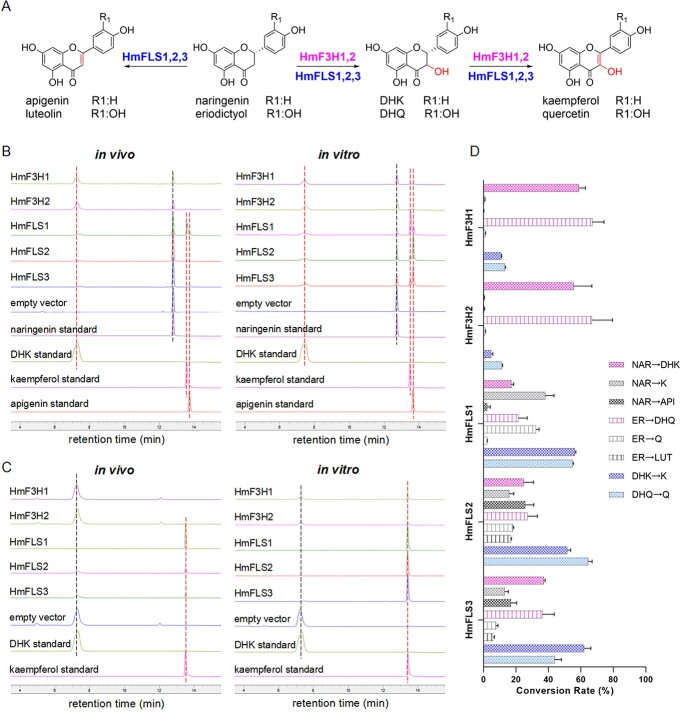
*In vivo* and *vitro* activity comparison between HmF3Hs and HmFLSs with substrates naringenin and DHK. (A) Catalytic scheme of HmF3Hs and HmFLSs. (B) *In vivo* and *vitro* functional discrepancy between HmF3Hs and HmFLSs with naringenin as the substrate. (C) *In vivo* and *vitro* functional discrepancy between HmF3Hs and HmFLSs with DHK as the substrate. Black dashed lines: substrates; red dashed lines: products. Detection wavelength: 330 nm. (D) Conversion rates of HmF3Hs and HmFLSs in producing different products *in vitro*. NAR, naringenin; ER, eriodictyol; DHK, dihydrokaempferol; DHQ, dihydroquercetin; K, kaempferol; Q, quercetin; API, apigenin; LUT, luteolin. Values represent means of three independent experiments ± standard deviation.

HmF3Hs belong to the 2-ODD (2-oxoglutarate-dependent dioxygenase) family as flavanone 3-hydroxylases. They were respectively constructed into the pET28a(+) empty vector, and heterologously expressed in *E. coli* BL21(DE3) to characterize enzymatic functions (Fig. 2, Supplementary Data Figs S2 and S3). Substrate feeding experiments showed that HmF3H1 and HmF3H2 could catalyze naringenin into dihydrokaempferol (DHK) efficiently *in vivo*, whereas they could not convert eriodictyol into dihydroquercetin (DHQ) unless cofactors (ascorbate + α-glutaric acid) were added (Fig. 2B, Supplementary Data Fig. S3B). In *E. coli*, they could not transform dihydroflavonols into flavonols either. However, the *in vitro* reactions found that they acted as bifunctional enzymes (Fig. 2A), which could catalyze naringenin and eriodictyol into the corresponding dihydroflavonols (DHK and DHQ), as well as catalyzing DHK and DHQ into small amounts of kaempferol and quercetin, respectively (Fig. 2, Supplementary Data Figs S3, S8–S19 and S25). The *in vitro* production of flavones by HmF3H1–2 was too slight to be counted, though LC–MS/MS spectra demonstrated the existence of flavones (Supplementary Data Figs S18 and S19). Since the protein sequence similarity between HmF3H1 and HmF3H2 was high (91.54%), it was reasonable that they possessed nearly identical catalytic activity.

**Figure 3:**
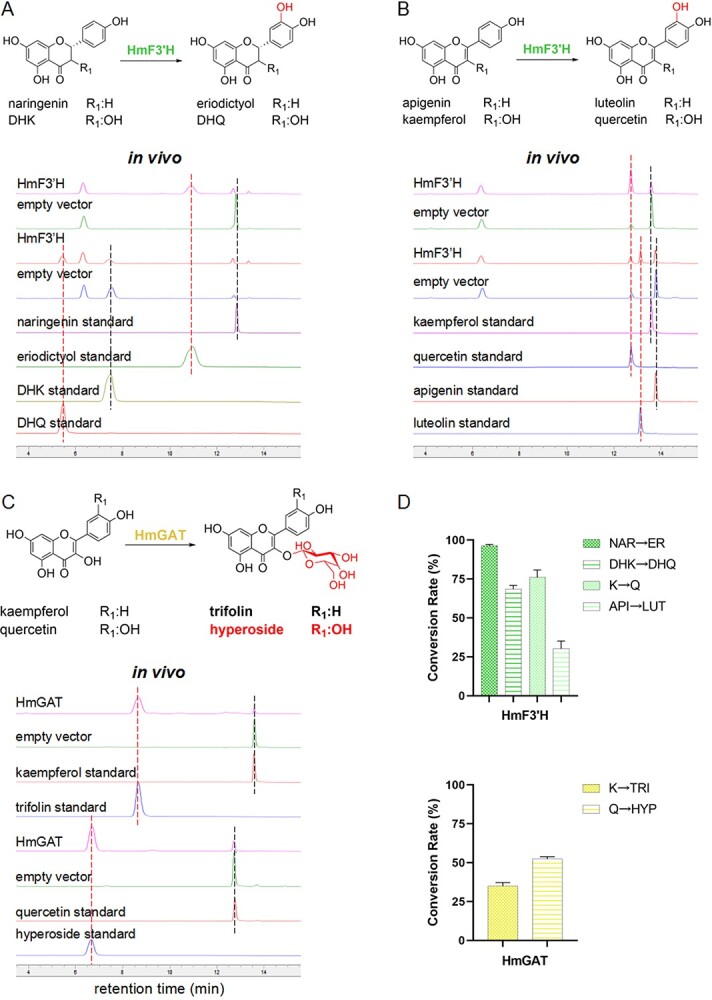
Functional characterization of HmF3′H and HmGAT*in vivo*. (A) *In vivo* activity of HmF3′H which catalyzed naringenin and DHK into eriodictyol and DHQ, respectively. Detection wavelength: 300 nm. (B) *In vivo* activity of HmF3′H which catalyzed kaempferol and apigenin into respective quercetin and luteolin. Detection wavelength: 360 nm. (C) *In vivo* verification of HmGAT, which catalyzed kaempferol and quercetin into corresponding flavonol 3-*O*-galactosides. Detection wavelength: 360 nm. Black dashed lines: substrates; red dashed lines: products. (D) Conversion rates of HmF3′H and HmGAT in producing different products *in vivo*. TRI, trifolin; HYP, hyperoside. Values represent means of three independent experiments ± standard deviation.

### HmFLSs have discrepant efficiencies in biosynthesizing flavonols

The same expression strategies were applied for HmFLS1–3 as were applied for HmF3Hs. Initially, we failed to characterize their *in vivo* activities. With the addition of cofactors (ascorbate + α-glutaric acid), they could convert dihydroflavonols into flavonols smoothly (Fig. 2C, Supplementary Data Fig. S3C). It was apparent that HmFLS2–3 could only produce small amounts of flavonols from dihydroflavonols *in vivo* (Fig. 2C, Supplementary Data Fig. S3C). However, HmFLS1 exhibited prominent power in converting dihydroflavonols, which could even catalyze the one-step production of flavonols and flavones by just feeding flavanones in *E. coli* (Fig. 2B, Supplementary Data Fig. S3B). The *in vitro* reactions further revealed the multifunctional roles of HmFLS1–3 (Fig. 2, Supplementary Data Fig. S3), which, in addition to catalyzing DHK and DHQ into kaempferol and quercetin, could convert flavanones into the corresponding dihydroflavonols, flavonols, and flavones. The LC–MS/MS analysis unveiled two distinct epimerized DHQ intermediates, namely (2*R*,3*R*)-*trans*-DHQ and (2*R*,3*S*)-*cis*-DHQ produced from eriodictyol (Fig. S3B, Supplementary Data Figs S20–S22). These intermediates exhibited the same [M + H]^+^ and fragmentation patterns, which were supposed to undergo α-face-attacking and β-face-attacking respectively by HmFLSs to produce flavonols and flavones [19, 20]. Furthermore, it was obvious that HmFLS1 mainly transformed flavanones into flavonols, while HmFLS2–3 (with 95.48% sequence similarity) favored the synthesis of flavones (Fig. 2D). Correspondingly, HmFLS1 had relatively low sequence similarity to HmFLS2–3 (48.05% and 46.85%, respectively), indicating two distinct branches in the phylogenetic course.

Since *in vivo* activities of HmFLSs entailed the addition of cofactors, we then investigated the effect of different cofactor combinations on the *in vivo* activities of HmFLSs and found that the addition of Fe^2+^ would reduce the production of flavonols substantially, whereas ascorbate plus α-glutaric acid were the best combination in prompting the production of flavonols by HmFLSs (Supplementary Data Figs S4 and S5). Despite this, Fe^2+^ positively promoted the *in vitro* activities of HmFLSs and HmF3Hs, where ascorbate and α-glutaric acid were consistently the functional determinants (Supplementary Data Fig. S6).

**Scheme 1:**
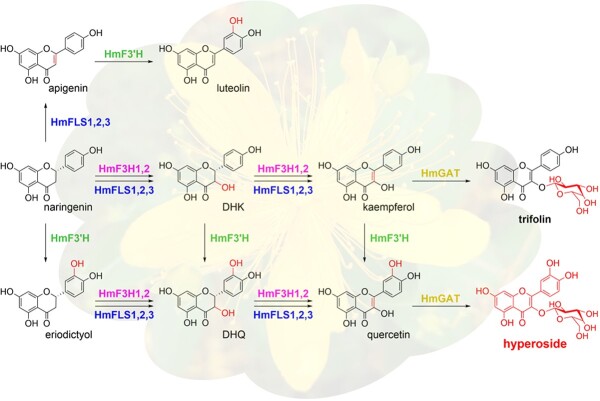
Hyperoside biosynthesis routes from naringenin in the flower buds of *H. monogynum*. DHK, dihydrokaempferol; DHQ, dihydroquercetin.

### HmF3′H acts broadly on 4′-hydroxyl flavonoids

HmF3′H belongs to the CYP450 enzyme family as a membrane protein that catalyzes the 3′-hydroxylation of flavonoids. To identify the function of HmF3′H, its coding sequence was cloned into the pYES2 plasmid and induced expression in the *WAT11* yeast strain containing *Arabidopsis thaliana* CYP450 reductase 1 (AtCPR1). Both *in vivo* and *in vitro* assays demonstrated that HmF3′H could catalyze naringenin, DHK, kaempferol, and apigenin into eriodictyol, DHQ, quercetin, and luteolin, respectively (Fig. 3A and B, Supplementary Data Figs S7, S23 and S25). Particularly, HmF3′H exhibited much higher catalytic efficiency towards naringenin, DHK, and kaempferol (Fig. 3D). Experiments also revealed that HmF3′H could not utilize baicalein or kaempferol 3-*O*-glycosides to produce the corresponding 3′-hydroxylated flavonoids, neither could HmF3′H catalyze the conversion of eriodictyol, DHQ, quercetin, or luteolin into 5′-hydroxylated products. These results indicated the specific role of HmF3′H in 3′-hydroxylation of 4′-hydroxyl flavonoids including flavanones, dihydroflavonols, flavonols, and flavones.

### HmGAT takes charge of the last step in hyperoside biosynthesis

HmGAT was initially annotated as a flavonoid 3-*O*-glycosyltransferase, while the conserved 3′-end His residue in the PSPG box relocated the enzyme as a galactosyltransferase (Supplementary Data Fig. S8) [21]. To better understand its function, *HmGAT* was cloned into the synthesized vector pCDFDuet1-BbGalE2 (*Bifidobacterium bifidum* UDP-glucose 4-epimerase 2, enabling the conversion of UDP-glucose into UDP-galactose). It was identified in *E. coli* to have strong 3-*O*-galactosyltransferase activities towards kaempferol and quercetin (Fig. 3C and D), suggesting it as a key enzyme for hyperoside biosynthesis in *H. monogynum*. However, it could not install the sugar moiety into dihydroflavonols (DHK and DHQ). The *in vitro* results (Supplementary Data Figs S9 and S24–S25) were consistent with the *in vivo* results, further solidifying that HmGAT accepts quercetin as the last step in hyperoside biosynthesis.

Thus far, we have successfully identified four kinds of enzymes needed for hyperoside biosynthesis in *H. monogynum*. Broad substrate selectivity of these enzymes implied parallel hyperoside biosynthesis routes in this plant (Scheme 1), which still needs experimental verification botanically in the future.

### Functional discrepancy analysis of HmF3Hs and HmFLSs by molecular docking and site-directed mutagenesis

**Figure 4:**
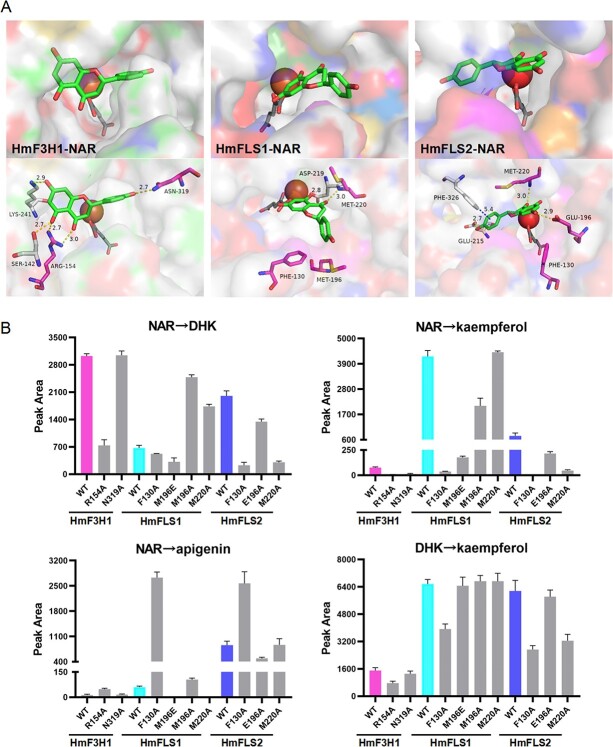
Characterization of new key residues ensuring catalytic activities of HmF3H1 and HmFLS1–2 by molecular docking and site-directed mutagenesis. (A) Molecular docking results of HmF3H1 and HmFLS1–2 with naringenin. NAR, naringenin; dark gray compound, α-glutaric acid. Yellow dashed lines: H-bond interactions. Blue dashed lines: π–π stacking interactions. Magenta residues: new conserved amino acid residues. (B) Activity variations of site-directed mutants HmF3H1 and HmFLS1–2. 40 mg crude enzymes and 0.2 mM substrates (NAR or DHK) were used for *in vitro* reactions (30°C, 350 rpm, 1.5 h). Values represent means of three independent experiments ± standard deviation.

Since overlapped functions were shown among HmF3Hs and HmFLSs which could also produce flavones (Fig. 2A), we conducted molecular docking and site-directed mutagenesis experiments with substrates naringenin and DHK to investigate the key residues involved in functional diversity and activity discrepancy of these enzymes. Molecular docking results explained the extremely high vigor of HmFLS1 in producing flavonols by generating a more open binding pocket (Fig. 4A, Supplementary Data Fig. S12). In combination with multi-sequence alignment results, the enzyme–substrate docking also preliminarily revealed new conserved residues for F3Hs (R154, N319, numbered on HmF3H1) and FLSs (F130, M(L)220, numbered on HmFLS1) by forming H-bond interactions or π–π stacking interactions with the two ligands (Fig. 4A, Supplementary Data Figs S10–S13).

To verify the key role of these newly identified conserved residues, site-directed mutagenesis experiments were conducted on HmF3H1 and HmFLS1–2 (Fig. 4B). Crude enzyme assays showed that residue R154 was important for the catalytic activity of HmF3H1, while N319 hardly affected the functions of HmF3H1. For HmFLS1, M196 played a crucial role in converting the substrate naringenin into kaempferol while it hardly affected the utilization of substrate DHK. M220 would restrain the production of DHK, whereas it did not impact kaempferol production. Above all, F130 was vital for HmFLS1 in converting both naringenin and DHK into kaempferol. It is interesting to note that F130A boosted the production of apigenin exponentially by prompting the full conversion of naringenin into apigenin rather than kaempferol. Similar influence patterns of F130 were also observed in HmFLS2, emphasizing its critical role in determining whether naringenin would flow into kaempferol or apigenin as a crucial functional boundary signal. In addition to F130, E196 and M220 also played important roles in naringenin utilization by HmFLS2. Specifically, E196 would help naringenin flow into apigenin while M220 would induce naringenin more into DHK and kaempferol. It is therefore reasonable that E196A hardly affected the conversion of DHK to kaempferol by HmFLS2, whereas M220A and F130A inhibited this conversion markedly. Finally, by combination of molecular docking results, multi-sequence alignment, and site-directed mutagenesis experiments, we could infer that the newly identified conserved residues R154 and F130 were respectively crucial for the activity of F3Hs and FLSs, while M220 contributed to flavonol production specifically catalyzed by HmFLS2–3. Meanwhile, E196 was important for HmFLS2–3 to produce flavones, which was also reported on CsFLS2 [22].

### *Escherichia coli* BL21(DE3) engineering for hyperoside production from naringenin

During the functional verification of HmGAT in *E. coli* BL21(DE3), we observed a substantial decrease in hyperoside content over time (Supplementary Data Fig. S14). However, the addition of cofactors (ascorbate + α-glutaric acid) could inhibit its decrease to some extent, though there was no known relationship between galactosyltransferases and these cofactors, which were normally needed for 2-ODD family enzymes. Drawing from previous studies [23–25] and the experimental results in this study (Supplementary Data Fig. S15A and B), we speculated that yhhW (a pirin-like protein that catalyzes the decomposition of flavonols into depsides and CO in *E. coli* for toxicity abatement) mediated endogenous decomposition of flavonoids in *E. coli* BL21(DE3) and Rossetta(DE3), among which 3′,4′-dihydroxylated flavonoids were degraded most severely. Given that we failed in knocking out *yhhW* from *E. coli* BL21(DE3), we then attempted to inhibit its activity with different cofactors. It turned out that the cofactor combination (5 mM ascorbate +5 mM α-glutaric acid) was the most effective in inhibiting flavonoid decomposition (Supplementary Data Fig. S15C), which would be considered in subsequent hyperoside-biosynthetic *E. coli* engineering.

The high vigor of HmFLS1 in converting dihydroflavonols and flavanones into flavonols, together with its FPKM change similarity to *HmGAT*, denoted the essential role of HmFLS1 in hyperoside biosynthesis in *H. monogynum* flower buds. Therefore, we chose HmFLS1 for subsequent pathway reconstruction in *E. coli* BL21(DE3).

**Figure 5:**
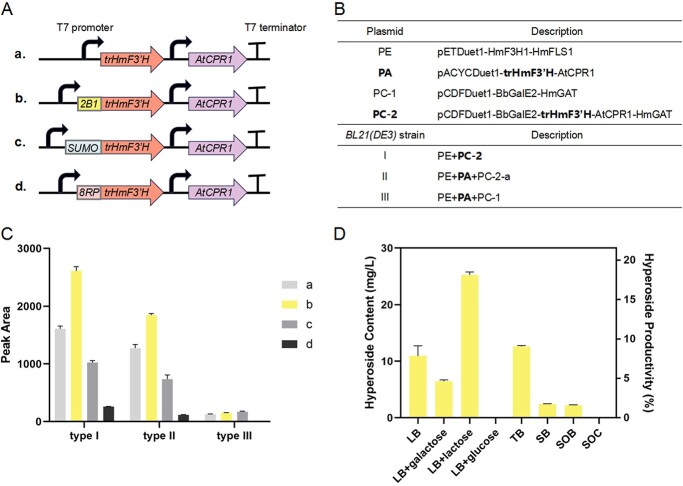
Hyperoside biosynthetic pathway reconstruction in *E. coli* BL21(DE3). (A) Short hydrophilic peptides (2B1, SUMO, and 8RP) were respectively inserted at the N terminal of trHmF3′H to enhance the solubility of trHmF3′H. (B) *E. coli* BL21(DE3) strain types with different co-expression vector combinations. **PA**, **PC-2**: recombinant vectors carrying one of the trHmF3′H variants shown in (A). (C) Hyperoside production in different recombinant *E. coli* BL21(DE3) strains. The strains were fed with 0.2 mM naringenin plus 5 mM cofactors (ascorbate + α-glutaric acid) for 15 h after 5 h IPTG (1 mM) induction. (D) Hyperoside yield of type I-b *E. coli* strain in different culture media. The strain was fed with 0.3 mM naringenin plus 5 mM cofactors (ascorbate + α-glutaric acid) for 6 h after 20 h IPTG (1 mM) induction along with 5 mM cofactors. trHmF3′H: HmF3′H whose N-terminal transmembrane signal peptide was truncated; BbGalE2: *B. bifidum* UDP-glucose 4-epimerase 2; AtCPR1: *A. thaliana* CYP450 reductase 1. Values represent means of three independent experiments ± standard deviation.

In reference to a previous study [26], we chose three co-expression vectors (pETDuet1, pCDFDuet1, pACYCDuet1) in different combinations to realize the co-expression of *HmF3H1*, *HmFLS1*, *trHmF3′H* (N-terminal-truncated HmF3′H), *AtCPR1* (*Arabidopsis thaliana* CYP450 reductase 1), *BbGalE2*, and *HmGAT* in *E. coli* BL21(DE3) (Fig. 5B), where each gene had its own T7 promoter and RBS site. To promote the soluble expression of trHmF3′H, different short hydrophilic peptides (including 2B1, SUMO, and 8RP) were respectively inserted at its N terminal (Fig. 5A). Consequently, type I recombinant *E. coli* strains were found to produce more hyperoside than type II and type III, among which type I-b showed the best performance (Fig. 5C, Supplementary Data Fig. S16). The feeding conditions for the type I-b strain were also optimized to achieve a higher yield of hyperoside (Fig. 5D, Supplementary Data Fig. S17). As a result, LB liquid medium supplemented with 20 mM lactose, 20 h IPTG (isopropyl β-d-thiogalactoside, 1 mM) induction along with 5 mM cofactors (ascorbate + α-glutaric acid), followed by 6 h feeding of 0.3 mM naringenin plus 5 mM cofactors, was found to produce hyperoside mostly at 25 mg/l (equivalent to 18.34% productivity) (Fig. 5D). In the end, the recombinant *E. coli* BL21(DE3) strain (Supplementary Data Fig. S16), which produced hyperoside by feeding naringenin, not only verified one of the hyperoside biosynthesis pathways elucidated in this study, but also demonstrated a successful attempt to obtain target compounds by biosynthetic pathway reconstruction and conditional optimization in heterologous strains.

**Figure 6:**
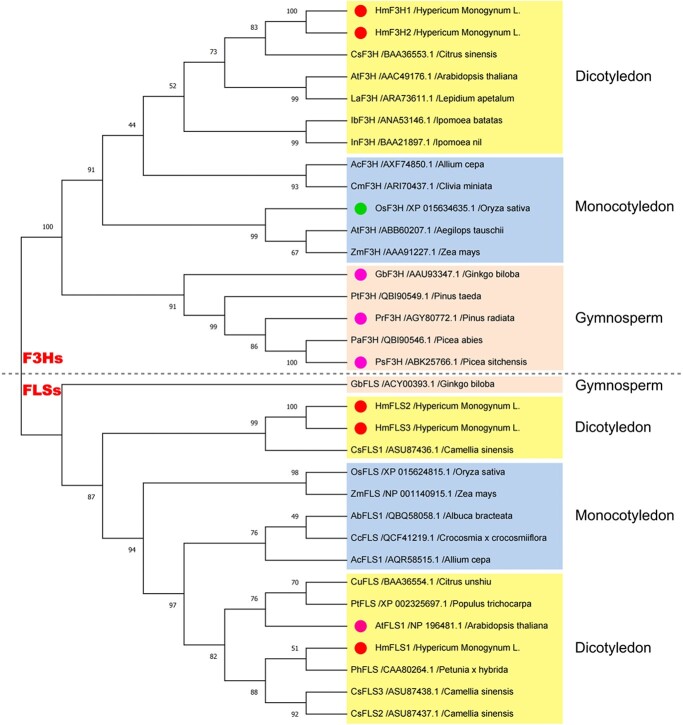
Phylogenetic tree of plant F3Hs and FLSs. Numbers on the nodes represent confidence percentages. The maximum likelihood method was applied and the bootstrap value was set as 1000. OsF3H in a green dot: with additional FLS activity; GbF3H, PrF3H, PsF3H, and AtFLS1 in magenta dots: with additional FNSI activity; HmF3H1–2 and HmFLS1–3 in red dots: with F3H, FLS and FNSI activities identified in this study.

## Discussion

### Evolutionary relationships among flavanone 3-hydroxylases, flavone synthases I, flavonol synthases, and anthocyanidin synthases

The 2-ODD family is a large cytosol-soluble enzyme family catalyzing various oxidation reactions, which can be divided into three classes (DOXA, DOXB, DOXC) in plants based on the sequence similarity and functions [27]. DOXA class proteins normally catalyze the oxidative demethylation of alkylated nucleic acids and histones, DOXB class proteins catalyze proline 4-hydroxylation in cell wall protein synthesis, and DOXC class proteins are involved in secondary metabolism of phytochemicals such as phytohormones and flavonoids [27]. F3Hs and FLSs both belong to DOXC-class proteins, which include FNSIs (flavone synthases I) and ANSs (anthocyanidin synthases) as well. About the evolutionary relationships among these four types of flavonoid-relevant 2-ODD enzymes, recent studies suggested that F3Hs evolved from FNSIs, while FNSIs, FLSs, and ANSs evolved independently from a common ancestor [20, 28].

In this study, though HmF3Hs belonged to the F3H family and HmFLSs belonged to the FLS family in the phylogenetic tree (Fig. 6), they all performed multifunctionally with varying catalytic efficiencies (Fig. 2A). The two different phylogenetic branches between HmFLS1 and HmFLS2–3 (Fig. 6) implied that HmFLS1 may evolve much later with more specific activity in biosynthesizing flavonols than HmFLS2–3, which produced plentiful flavones as well. Previous studies found that OsF3H could catalyze dihydroflavonols into flavonols [29]; GbF3H, PrF3H, and PsF3H could catalyze naringenin into DHK and slight amounts of apigenin [28]; AtFLS1, CsFLS2, and AtANS could catalyze naringenin into DHK, kaempferol and apigenin [22, 30, 31]. These phenomena implied a deeper connection among F3Hs, FNSIs, FLSs, and ANSs – that the four types of 2-ODD had overlapped functions with each other in different degrees by evolving from a mutual gene or possibly two isozyme genes, which were partly silent or activated through mutation during plant evolution. More explorations about F3Hs, FLSs, FNSIs, and ANSs from incipient plant species will be needed to further reveal the evolutionary relationships among the four types of 2-ODD enzyme.

### Cofactors needed for flavonol synthases and flavanone 3-hydroxylases

The 2-ODD enzymes typically require ascorbate (as an activator and iron-reducing agent), Fe^2+^ (as a cofactor), O_2_ and α-glutaric acid (as co-substrates) to exert functions [20, 27, 32]. The functional verification of FLSs is normally conducted *in vitro* with all cofactors added. Ascorbate and α-glutaric acid were reported to be necessary and a low concentration of Fe^2+^ to be ancillary [33–35]. However, it was rarely reported about the characterization of FLSs in *E. coli* cultures except for one that successfully characterized FLS activities *in vivo* by adding substrates together with cofactors [36].

To begin with, we failed to conduct *in vivo* verification of HmFLSs with no cofactors added. Later, with all cofactors (ascorbate, α-glutaric acid, Fe^2+^) added, HmFLS-recombinant *E. coli* strains succeeded in synthesizing flavonols from dihydroflavonols. Furthermore, ascorbate and α-glutaric acid were found to be vital in producing flavonols *in vivo* while Fe^2+^ played a negative role in it (Supplementary Data Figs S4 and S5). The possible reason might be that LB liquid medium already contained adequate amounts of Fe^2+^ (1–10 μM) for 2-ODD function [37], and the extra addition of Fe^2+^ into *E. coli* cultures would cause the formation of water-insoluble iron–flavonoid complexes as well as enhanced decomposition activity mediated by yhhW [24, 38], thus leading to the vanishing of some kinds of flavonoids in *E. coli*. Therefore, the *in vivo* addition of extra Fe^2+^ was unnecessary and detrimental. The flavonols with 3′,4′-OH were also found to be unstable in the medium or solution [39], which could be one of the reasons for flavonoid vanishing. One study [40] found that the bifunctional OcFLS (*Ornithogalum caudatum*) would exert different functions with or without Fe^2+^*in vitro*. In our study, Fe^2+^ played a positive but non-essential role during the *in vitro* verification of HmF3Hs and HmFLSs (Supplementary Data Fig. S6). Hence, we speculate that Fe^2+^ has diverse effects on FLSs and F3Hs from different species, while ascorbate and α-glutaric acid are consistently necessary for the functional verification of 2-ODDs, especially for the *in vivo* activity of FLSs, as confirmed in our study.

### Biological roles of hyperoside in flower buds and the selection of a heterologous biosynthesis system

In the flower buds of *H. monogynum*, hyperoside was produced the most at Lev. 2 stage and in the petals of the Lev. 4 stage, indicating its significant role in reproductive growth, chemical defense, petal color, and pollinator attraction. In previous studies, hyperoside was found to promote pollen tube growth by regulating the depolymerization effect of actin-depolymerizing factor 1 on microfilaments in okra [6]. It was also found to stimulate its own biosynthesis via the CDPK6-MYB30-GAT signaling pathway, which in turn, prompted the pollen fertility and seed set of okra by prolonging flower duration and promoting pollen germination [7, 8]. The positive influence of exogenous hyperoside on flower buds indicates its great prospects in horticulture and agriculture applications. A recent study reported that sorbitol could promote the accumulation of hyperoside and ultimately prompt loquat flower bud differentiation through ERF12-CAL-GAT signaling pathway, again demonstrating the significant role of hyperoside in flower bud formation by potentially influencing downstream genes [41]. Therefore, it is of great significance to synthesize hyperoside in an efficient way.

For the heterologous biosynthesis of hyperoside, three kinds of model system (plants, yeast, *E. coli*) could be selected, in which tobacco and *A. thaliana* were usually the first choice for plant-derived enzyme verification with much closer phylogenetic relationships. Previous studies reported that transient co-expression of most montbretin A biosynthesis genes in *Nicotiana benthamiana* resulted in the production of 2 mg/g fresh weight of myricetin 3-*O*-glucosyl rhamnoside [42]; and *MrF3′5′H*-transgenic *Nicotiana tabacum* could produce 0.15 mg/g fresh weight of myricetin rutinoside [43]. In *Saccharomyces cerevisiae*, the daidzin biosynthesis pathway was reconstructed with productivity of 73.2 mg/l [44]; kaempferol and quercetin biosynthesis pathways were reconstructed with productivity of 26.57 ± 2.66 mg/l and 20.38 ± 2.57 mg/l, respectively [45]. In *E. coli* BL21(DE3), baicalein and scutellarein biosynthesis pathways were engineered with productivity of 23.6 mg/l and 106.5 mg/l, respectively [26]; meanwhile, transforming AtRHT into engineered *E. coli* W led to the production of 1.12 g/l quercitrin [46]. Since *E. coli* systems were much more direct, convenient, and less influenced by environmental factors, and pathway reconstruction was also much more adjustable and efficient in *E. coli* with short reproductive cycles, we decided to use *E. coli* BL21(DE3) for hyperoside biosynthesis pathway reconstruction.

### *Escherichia coli* engineering for heterologous biosynthesis of hyperoside

In this study, the *yhhW* gene was identified in the commercialized *E. coli* BL21(DE3) strain through reported genome information (GenBank: CP001509.3 [25]) and PCR cloning, and the enzyme activity was prominently inhibited by cofactors (ascorbate + α-glutaric acid) (Supplementary Data Fig. S15). To obstruct the degradation of flavonoids and boost hyperoside productivity, 5 mM cofactors (ascorbate + α-glutaric acid) were added twice to the type I-b recombinant *E. coli* strain during induction. The cell factory showed an ordinary hyperoside yield at 25 mg/l by feeding naringenin (Fig. 5D), which was much lower than the engineered *E. coli* W strain reported before [46], with the yield at 0.94 g/l by feeding quercetin. Other engineered *E. coli* strains were also reported with hyperoside yields at 0.28 g/l and 0.83 g/l by feeding quercetin in batches [47, 48]. One study investigated the one-pot *in vitro* synthesis of hyperoside by a three-enzyme cascade with batched quercetin substrate, and achieved the yield of 2.13 g/l [49]. To our knowledge, our study is the first report of an engineered *E. coli* strain utilizing inexpensive naringenin to produce hyperoside, which indicated the successful reconstruction of the hyperoside–biosynthetic pathway in *E. coli*. However, the continuous decomposition of flavonoids by yhhW constrained hyperoside productivity from reaching a higher level. Knocking out *yhhW* in commercialized *E. coli* strains would be favorable for boosting hyperoside productivity substantially as well as benefiting flavonoid-relevant enzyme research in *E. coli*.

In conclusion, this research revealed the parallel hyperoside biosynthesis routes potentially existing in the flower buds of *H. monogynum*, which expanded our comprehension of the corresponding plant enzyme families significantly. Pathway reconstruction in *E. coli* not only provided a new way to obtain hyperoside but uncovered its remolding potential by knocking out *yhhW*. Furthermore, other than FNSIIs and FNSIs, the multifunctional role of HmFLS2–3 implied a third inconspicuous path towards flavone production and accumulation in plants.

## Materials and methods

### Plant material, RNA extraction, and cDNA synthesis

*Hypericum monogynum* plants were planted as ornamental shrubs at China Pharmaceutical University, Nanjing, People’s Republic of China. The species was identified by Professor Mian Zhang (China Pharmaceutical University). *H*.* monogynum* flower buds at different stages (Lev. 1–4, without calyx) together with different tissues (stamens, pistils, petals) of Lev. 4 were collected for transcriptome database construction (Novogene, China). RNA was extracted using the E.Z.N.A. Plant RNA Kit (Omega, America), and first-strand cDNA synthesis was accomplished using the HiScript III 1st Strand cDNA Synthesis Kit (Vazyme, China).

### Screening and cloning of candidate genes

Candidate genes (*HmF3Hs*, *HmFLSs*, *HmF3′H*, *HmGAT*) were obtained by local BLASTP in the *H. monogynum* transcriptome database (see Data availability section), combined with transcriptome annotations from various public databases (GO, KEGG, KOG, Nr, Nt, Pfam, Swiss-Prot) and the analysis of conserved amino acid residues. Primers with specific restriction sites and plasmid homologous arms (Supplementary Data Table S1) were designed for PCR cloning on the first-strand cDNA. The resulting gene products were individually cloned into the empty vector pET28a(+) (for *HmF3Hs* and *HmFLSs*), pCDFDuet1-BbGalE2 (for *HmGAT*), or pYES2 (for *HmF3′H*) using the One Step Cloning Kit (Vazyme, China), followed by transformation into *E. coli* DH5α competent cells according to the protocol. Recombinant plasmids were extracted (E.Z.N.A. Endo-free Plasmid Mini Kit II, Omega, USA) for sequencing.

### Analysis of gene transcription levels and hyperoside metabolic levels

Transcript heat maps were constructed using OmicShare tools (www.omicshare.com/tools) through *Z*-score normalization of FPKM values. Flavonoids were extracted according to reported procedures [50] with some modifications. Flower buds of Lev. 1–4 (without calyx) and stamens, petals, and pistils of Lev. 4 were individually harvested, frozen, and ground into fine powder under liquid N_2_, followed by immediate lyophilization. Then, the lyophilized samples were immersed in 70% methanol (40 mg/ml) at 4°C for 24 h. After centrifugation at 12 000 rpm for 5 min, the supernatants were filtered through 0.22 μm organic filter membranes for HPLC analysis.

### Heterologous expression of HmF3Hs, HmFLSs, and HmGAT in *E. coli*

The successfully constructed recombinant vectors (pET28a(+)-HmF3Hs, pET28a(+)-HmFLSs, and pCDFDuet1-BbGalE2-HmGAT) were respectively transformed into *E. coli* BL21(DE3) competent cells. Positive colonies were selected and cultured in LB liquid medium with the corresponding antibiotic at 37°C, 180 rpm. When OD_600_ reached 0.5–0.7, IPTG (final concentration 0.5 mM) was added to induce protein expression (20 h, 150 rpm). Proteins were purified and enriched using BeyoGold™ His-Tag Purification Resin (Beyotime, China) for subsequent SDS–PAGE analysis and *in vitro* enzyme assays. *In vivo* assays were conducted by 12 h feeding of 0.1 mM substrates plus cofactors (2.5 mM sodium ascorbate + 2.5 mM α-glutaric acid) after 5 h IPTG induction. The products from 2 ml *E. coli* cultures were extracted by adding the equivalent volume of ethyl acetate twice. The organic layers were collected and evaporated to dryness in vacuum, then 200 μl methyl alcohol was added to fully dissolve the solid mixture, which was followed by filtration through a 0.22 μm organic filter membrane and centrifugation at 12 000 rpm for 10 min. The supernatants were subjected to HPLC analysis.

### Heterologous expression of HmF3′H in *Saccharomyces cerevisiae*

The *WAT11* yeast strain harboring AtCPR1 was selected as the host of the recombinant vector pYES2-HmF3′H. Transformation was accomplished with the FROZEN-EZ Yeast Transformation II Kit (Zymo Research, USA). Positive colonies were further cultured in liquid SD-U medium (containing 2% glucose) at 30°C, 200 rpm. When OD_600_ reached 0.5–0.7, cells were centrifuged at 4000 rpm for 5 min and washed twice with ddH_2_O. Then the cell precipitates were resuspended in liquid induction medium (containing 2% galactose) and protein expression was induced for 36 h at 28°C, 160 rpm. The microsomal proteins were extracted according to the reported protocol [51] for subsequent *in vitro* enzyme assays. *In vivo* enzyme assays were conducted by feeding 0.1 mM substrates and culturing for 48 h after 5 h protein induction. The products from 2 ml *S. cerevisiae* cultures were extracted by adding the equivalent volume of ethyl acetate twice. The organic layers were collected and evaporated to dryness in vacuum, then 200 μl methyl alcohol was added to fully dissolve the solid mixture for HPLC analysis.

### *In vitro* enzyme assays

For 2-ODD enzymes (HmF3Hs, HmFLSs), *in vitro* reaction conditions (per 1 ml volume) were set as follows: 100 mM NaH_2_PO_4_ (pH 6.5), 10 mM α-glutaric acid, 10 mM sodium ascorbate, 0.25 mM FeSO_4_, 0.2 mM DMSO-dissolved substrates, and 300 μg purified protein, incubating at 30°C, 300 rpm for 2 h. For HmGAT, *in vitro* reaction conditions (per 1 mL volume) were as follows: 50 mM Tris–HCl buffer (pH 7.5), 0.5 mM sugar donor, 0.2 mM DMSO-dissolved substrates, and 600 μg crude protein, incubating at 30°C, 300 rpm for 2 h. For CYP450 enzyme HmF3′H, *in vitro* reaction conditions (per 1 mL volume) were set as follows: 50 mM Tris–HCl buffer (pH 7.5), 1 mM NADPH, 0.2 mM DMSO-dissolved substrates, and 1 mg microsomal protein, incubating at 30°C, 300 rpm for 6 h.

Reactions were terminated by adding ethyl acetate (1 ml, twice). The organic layers were collected and evaporated to dryness in vacuum, then 200 μl methyl alcohol was added to fully dissolve the solid mixture for HPLC and LC–MS/MS analysis.

### Biosynthetic pathway reconstruction in *E. coli*

*HmF3H1* and *HmFLS1* were sequentially cloned into the co-expression vector pETDuet1; the restriction sites were SacI/NotI and XhoI, respectively.* HmGAT* was cloned into the synthesized co-expression vector pCDFDuet1-BbGalE2; the restriction site for *HmGAT* was XhoI. Then, T7-RBS-*trHmF3′H* (forms a–d, Fig. 5A) and T7-RBS-*AtCPR1* were both synthesized into the recombinant vector pCDFDuet1-BbGalE2-HmGAT; the restriction sites were AscI/ClaI and ClaI/NotI, respectively. T7-RBS-*trHmF3′H* (forms a–d, Fig. 5A) and T7-RBS-*AtCPR1* were also sequentially cloned into the third co-expression vector pACYCDuet1; the restriction sites were BamHI and XhoI, respectively. The primers involved were listed in Supplementary Data Table S2. Then, these recombinant vectors were transformed into *E. coli* BL21(DE3) competent cells in various combinations. Such culture conditions as IPTG concentration, protein-induction duration, naringenin concentration, substrate-feeding duration, cofactor kinds and concentration, and liquid medium kinds were explored to obtain the highest yield of hyperoside.

### Protein sequence analysis and phylogenetic tree construction

The protein sequences of plant GATs, HmF3Hs, and HmFLSs were subjected to multiple sequence alignment and analyzed by DNAMAN. The theoretical molecular weights of the corresponding proteins were calculated using the online analysis tool (https://www.expasy.org/resources/compute-pi-mw). Phylogenetic trees of plant F3Hs, FLSs, and GATs were respectively constructed by MEGA X (see Data availability section). The maximum likelihood method was applied and the bootstrap value was set as 1000.

### Protein homologous modeling and molecular docking

The 3D protein models of HmF3Hs and HmFLSs were established by SWISS-MODEL (https://swissmodel.expasy.org/) [52] based on the AtANS crystal structure (PDB: 1GP4) [53]. Molecular docking with naringenin and DHK was conducted using the software Maestro-Schrodinger [54]. The general procedures included ligand preparation, protein preparation, receptor grid generation, and ligand docking. Default parameters were adopted except for the setting of ligand-centered 20-Å grid boxes, rotatable groups, and extra precision. The molecular docking results were displayed and analyzed by Maestro-Schrodinger for diagrams as well as PyMOL for 3D views.

### Site-directed mutagenesis of HmF3H1 and HmFLS1–2

Site-directed mutagenesis of the three enzymes was conducted by using mutated primers for PCR cloning on the corresponding templates pET28a(+)-HmF3H1, pET28a(+)-HmFLS1, and pET28a(+)-HmFLS2. Primers designed for the experiment are listed in Supplementary Data Table S3. The cloning products were purified and ligated according to the protocol of Mut Express II Fast Mutagenesis Kit V2 (Vazyme, China). The recombinant products were respectively transformed into *E. coli* DH5α for sequencing. Then, correct plasmids were extracted and transformed into *E. coli* BL21(DE3) to induce protein expression. When the OD_600_ of *E. coli* cultures reached around 0.5, a final concentration of 0.5 mM IPTG was added and *E. coli* cultures were cultivated at 150 rpm, 20°C for 17 h. Proteins were extracted by ultrasonication, then filtered through 0.45 μm membranes. The reaction conditions (per 1 ml volume) for each well were as follows: 40 mg crude protein, 15 mM sodium ascorbate, 15 mM α-glutaric acid, 0.25 mM FeSO_4_, 50 mM NaH_2_PO_4_ (pH 6.5), 0.2 mM substrate (naringenin or DHK), incubating at 30°C, 350 rpm for 1.5 h. Reactions were terminated by adding ethyl acetate (1 ml, twice). The organic layers were collected and evaporated to dryness in vacuum, then 200 μl methyl alcohol was added to fully dissolve the solid mixture for HPLC analysis.

### HPLC and LC–MS/MS analysis

HPLC analysis was conducted on an Agilent 1290 Series HPLC System (Agilent, USA), and the mobile phases were as follows: 0.1% formic acid in ultrapure water as A, and chromatographically pure methanol as B. Elution conditions were as follows: 0–8 min, 50% B; 8–12 min, 50–98% B; 12–20 min, 98% B. The flow rate was 1 ml/min, column temperature was 30°C, and detection wavelengths were 270, 300, 330, and 360 nm.

LC–MS/MS spectra were determined in a Bruker Esquire-LC quadrupole ion trap mass spectrometer (Germany). HPLC conditions were the same as mentioned above with detection wavelength at 270 nm. The ESI source operated in both positive and negative modes; capillary voltage was set at 4500 V; end plate offset voltage was set at 500 V; nebulizer pressure was 20.0 psi; drying gas temperature was 200°C; drying gas (N_2_) flow rate was 4.0 l/min; and the collision gas was ultrapure N_2_. Samples were introduced into the electrospray source at a flow rate of 4 μl/min. Targeted MS/MS mode (*m*/*z* 250 ± 200) was used to analyze the top two ions. The scan rate was three spectra per second in the range of *m*/*z* 100–1000 for MS and *m*/*z* 100–1000 for MS/MS.

## Acknowledgements

This work was supported by the National Natural Science Foundation of China (No. 32070389), and the ‘Double First-Class’ University project of China Pharmaceutical University (CPU2022QZ29). We appreciate Dr Hindra from the Department of Biology at McMaster University for helping us organize the manuscript and improve the language.

## Author contributions

J.L., Y.W., and L.K. designed the research; Y.W. performed the experiments, and Z.C., Q.L., S.Z., Y.L., and X.L. assisted with the experiments; Y.W. wrote the manuscript; J.L. and L.K. revised the manuscript. All authors read and approved the final version of the manuscript.

## Data availability

All data related to this research are available in this paper and its supplementary materials published online.

GenBank accession numbers involved in this article are as follows:

Query proteins: OsFLS (XP_015624815.1); GbFLS (ACY00393.1); MtF3H (ACR15123.1); PhGAT (AAD55985.1); PhF3′H (AAD56282.1).

Phylogenetic tree of plant GATs: PhGAT (AAD55985.10); VmGAT (BAA36972.1); AgGAT (AXU98426.1); DkGAT (BAI40148.1); DcGAT (AKI23632.1); EgGAT (BAF49284.1); EkGAT (QTU90759.1); AcGAT (BAD06514.1); AcGAT (A0A2R6Q8R5.1); SbGT (QBL54224.1); FaGLT (Q2V6K0.1); GmGLT (NP_001304377.2); VvGLT (AAB81682.1); FhFLT (ADK75021.1); EkGLT (QTU90760.1); PfGLT (BAA19659.1).

Phylogenetic tree of plant F3Hs and FLSs: AbFLS1 (QBQ58058.1); AcFLS1 S(AQR58515.1); AtF3H (ABB60207.1); AcF3H (AXF74850.1); AtF3H (AAC49176.1); AtFLS1 (NP_196481.1); CcFLS (QCF41219.1); CmF3H (ARI70437.1); CsF3H (BAA36553.1); CsFLS1 (ASU87436.1); CsFLS3 (ASU87438.1); CsFLS2 (ASU87437.1); CuFLS (BAA36554.1); GbFLS (ACY00393.1); GbF3H (AAU93347.1); IbF3H (ANA53146.1); InF3H (BAA21897.1); LaF3H (ARA73611.1); OsF3H (XP_015634635.1); OsFLS (XP_015624815.1); PaF3H (QBI90546.1); PhFLS (CAA80264.1); PrF3H (AGY80772.1); PtF3H (QBI90549.1); PsF3H (ABK25766.1); PtFLS (XP_002325697.1); ZmF3H (AAA91227.1); ZmFLS (NP_001140915.1).

Sequence data of HmF3H1–2, HmFLS1–3, HmF3′H, and HmGAT identified in this study have been submitted to the GenBank database under accession numbers OP585373, OP585374, OP585375, OP585376, OP585377, OP585378, and OP585379, respectively. The transcriptome database of *H. monogynum* has been submitted to NCBI under project ID PRJNA907155 [55].

## Conflict of interest

The authors declare no conflicts of interest.

## Supplementary data

Supplementary data is available at *Horticulture Research* online.

## Supplementary Material

Web_Material_uhad166
