# Supplementary material for: The parallel biosynthesis routes of hyperoside from naringenin in Hypericum monogynum
Source: Hortic Res. 2023 Aug 17;10(9):uhad166. doi: 10.1093/hr/uhad166 (PMC10506691; doi:10.1093/hr/uhad166)
Supplement: Web_Material_uhad166 [file web_material_uhad166.zip › Supporting Information-clean.pdf]

# Supporting Information of

## The parallel biosynthesis routes of hyperoside from naringenin in *Hypericum monogynum*

**Yingying Wang, Zhirong Cui, Qianqian Li, Shuai Zhang, Yongyi Li, Xueyan Li, Lingyi Kong* and Jun Luo***

*Jiangsu Key Laboratory of Bioactive Natural Product Research and State Key Laboratory of Natural Medicines, Department of Traditional Chinese Pharmacy, China Pharmaceutical University, Nanjing 210009, China*

*Corresponding authors. E-mail: luojun@cpu.edu.cn; cpu_lykong@126.com.

**Table S1.** Gene cloning primers used in this study.

**Table S2.** Primers involved in multi-gene co-expression vector construction.

**Table S3.** Primers involved for site-directed mutagenesis on HmF3H1 and HmFLS1-2.

**Figure S1.** FPKM levels of hyperoside-biosynthetic relevant enzymes at different bud stages and tissues of Lev.4 flower buds from *H. monogynum*.

**Figure S2.** SDS-PAGE of $Ni^{2+}$ resin-purified proteins.

**Figure S3.** *In vivo* and *vitro* activity comparison between HmFLSs and HmF3Hs, with substrates eriodictyol and DHQ.

**Figure S4.** The influence of cofactors on HmFLSs catalyzing DHK into kaempferol *in vivo*.

**Figure S5.** The influence of cofactors on HmFLSs catalyzing DHQ into quercetin *in vivo*.

**Figure S6.** The influence of cofactors on HmF3Hs and HmFLSs during *in vitro* verification.

**Figure S7.** *In vitro* verification of HmF3′H with corresponding 3′-hydroxylated products.

**Figure S8.** The phylogenetic tree and multiple-sequence alignment result of plant GATs.

**Figure S9.** HmGAT functional verification *in vitro*, which catalyzed kaempferol and quercetin into trifolin and hyperoside, respectively.

**Figure S10.** The overall molecular docking results of HmF3H1 and HmFLS1-3 with NAR, respectively.

**Figure S11.** Multiple-sequence alignment result of HmF3Hs and HmFLSs.

**Figure S12.** Molecular docking results of HmF3Hs and HmFLSs with NAR and DHK, respectively.

**Figure S13.** The overall molecular docking results of HmF3Hs and HmFLSs with two substrate ligands (NAR, DHK) displayed in diagrams.

**Figure S14.** Hyperoside production in *E. coli* BL21(DE3) strain catalyzed by HmGAT with/without cofactors at different time points.

**Figure S15.** Flavonoids decomposition in *E. coli* mediated by yhhW.

**Figure S16.** The layout of engineered *E. coli* BL21(DE3) factory (type I-b) for producing hyperoside.

**Figure S17.** Hyperoside production in type I-b *E. coli* BL21(DE3) strain under different cofactors.

**Figure S18.** The LC-MS/MS spectra and UV chromatograms of *in vitro* catalytic products by HmF3H1 with four substrates.

**Figure S19.** The LC-MS/MS spectra and UV chromatograms of *in vitro* catalytic products by HmF3H2 with four substrates.
**Figure S20.** The LC-MS/MS spectra and UV chromatograms of *in vitro* catalytic products by HmFLS1 with four substrates.
**Figure S21.** The LC-MS/MS spectra and UV chromatograms of *in vitro* catalytic products by HmFLS2 with four substrates.
**Figure S22.** The LC-MS/MS spectra and UV chromatograms of *in vitro* catalytic products by HmFLS3 with four substrates.
**Figure S23.** The LC-MS/MS spectra and UV chromatograms of *in vitro* catalytic products by HmF3′H, with NAR, DHK, K, and API as respective substrate.
**Figure S24.** The LC-MS/MS spectra and UV chromatograms of *in vitro* catalytic products by HmGAT, with K and Q as respective substrate.
**Figure S25.** The LC-MS/MS spectra and UV chromatograms of the compound standards.

**Table S1.** Gene cloning primers used in this study.

| Primer | Sequence (5′ → 3′) | Plasmid | Restriction Sites |
|---|---|---|---|
| HmF3H1-F | cgcggatccgaattcgagctcGGATATCATTATCAACTCCAAAATCTACA | pET28a(+) | SacI/NotI |
| HmF3H1-R | tggtggtgctcgagtgcggccgcAGCAAAGATCTCATCAATGGGC | | |
| HmF3H2-F | cgcggatccgaattcgagctcATGGCTCCAACACCAAAAACG | pET28a(+) | SacI/NotI |
| HmF3H2-R | tggtggtgctcgagtgcggccgcAGCAAAGATCTCATCAATGGGC | | |
| HmFLS1-F | cgcggatccgaattcgagctcATGGAGGTGGCAAGGGTTCA | pET28a(+) | SacI/NotI |
| HmFLS1-R | tggtggtgctcgagtgcggccgcCTGTGGGAGCTTGTTGAGCTTG | | |
| HmFLS2-F | cgcggatccgaattcgagctcATGGAGGTGGAGAGAGTGCAAG | pET28a(+) | SacI/NotI |
| HmFLS2-R | tggtggtgctcgagtgcggccgcTTGAGGGAGCTTGTTGAATTTGC | | |
| HmFLS3-F | cgcggatccgaattcgagctcATGGAGGTGGAGAGAGTGCAAG | pET28a(+) | SacI/NotI |
| HmFLS3-R | tggtggtgctcgagtgcggccgcTTGAGGGAGCTTGTTGAATTTGC | | |
| HmF3′H-F | attaagcttggtaccgagctcATGTCTCCTTTTGTTCTGTACTCATTTG | pET28a(+) | SacI/NotI |
| HmF3′H-R | tagatgcatgctcgagcggccgcTTAATTTGAGGCCTTGTAGGCTTG | | |
| HmGAT-F | gctgacgtcggtaccctcgagATGGAGGAGAAAGGACACGTGG | pCDFDuet1 | XhoI |
| HmGAT-R | ggtttctttaccagactcgagCAATTTCTTGGTATTGGTCACAATC | | |

**Table S2.** Primers involved in multi-gene co-expression vector construction.

| Primer | Sequence (5′ → 3′) | Plasmid | Restriction Sites |
|---|---|---|---|
| trF3′H-F | tcatcaccacagccaggatccGATGTATAGTGGACGTCGTCTCCCG | pACYCDuet1 | BamHI |
| trF3′H-R | gccgagctcgaattcggatccTTAATTTGAGGCCTTGTAGGCTTG | | |
| 2B1-trF3′H-F | tcatcaccacagccaggatccGATGGCTAAGAAAACGAGCTCTAAAG | pACYCDuet1 | BamHI |
| 2B1-trF3′H-R | gccgagctcgaattcggatccTTAATTTGAGGCCTTGTAGGCTTG | | |
| SUMO-trF3′H-F | tcatcaccacagccaggatccGATGTCGGACTCAGAAGTCAATCAA | pACYCDuet1 | BamHI |
| SUMO-trF3′H-R | gccgagctcgaattcggatccTTAATTTGAGGCCTTGTAGGCTTG | | |
| 8RP-trF3′H-F | tcatcaccacagccaggatccGATGGCTCTGTTATTAGCAGTTTTTATG | pACYCDuet1 | BamHI |
| 8RP-trF3′H-R | gccgagctcgaattcggatccTTAATTTGAGGCCTTGTAGGCTTG | | |
| AtCPR1-F | gctgacgtcggtaccctcgagATGACCTCCGCGCTGTACG | pACYCDuet1 | XhoI |
| AtCPR1-R | ggtttctttaccagactcgagTTACCAAACATCACGCAGGTAGC | | |
| HmF3H1-F | ccaggatccgaattcgagctcAGGATATCATTATCAACTCCAAAATCTACA | pETDuet1 | SacI/NotI |
| HmF3H1-R | cgacttaagcattatgcggccgcTTAAGCAAAGATCTCATCAATGGG | | |
| HmFLS1-F | gctgacgtcggtaccctcgagATGGAGGTGGCAAGGGTTCA | pETDuet1 | XhoI |
| HmFLS1-R | ggtttctttaccagactcgagCTGTGGGAGCTTGTTGAGCTTG | | |
| yhhW-F | ATGATCTACTTACGCAAAGCAA | - | - |
| yhhW-R | TTAAACCGGCGGCAGATCGAAC | | |

**Table S3.** Primers involved for site-directed mutagenesis on HmF3H1 and HmFLS1-2.

| Primer | Sequence (5′ → 3′) | Plasmid | Restriction Sites |
|---|---|---|---|
| HmF3H1-R154A-F | AAGACTGGgcgGAGATAGTGACTTACTTCTCATACCCAAT | pET28a(+) | SacI/NotI |
| HmF3H1-R154A-R | TATCTCcgcCCAGTCTTGGACCGCCTCCCCCT | | |
| HmF3H1-N319A-F | ACATTCCAAgcgccaGCACCAGATGCAACTGTGTATCC | pET28a(+) | SacI/NotI |
| HmF3H1-N319A-R | TGCtggcgcTTGGAATGTGGCTATTGACAATCT | | |
| HmFLS1-F130A-F | CGATCATTTGgcgCATAAGATATGGCCGTCGGAA | pET28a(+) | SacI/NotI |
| HmFLS1-F130A-R | TATGcgcCAAATGATCGACCCAACCCTTCTTC | | |
| HmFLS1-M196E-F | GGAGTACgaaCTCAAGATTAATTTTTACCCACCTTG | pET28a(+) | SacI/NotI |
| HmFLS1-M196E-R | TCTTGAGttcGTACTCCAGTACTTTACCGCCGA | | |
| HmFLS1-M196A-F | GAGTACgcgCTCAAGATTAATTTTTACCCACCTTG | pET28a(+) | SacI/NotI |
| HmFLS1-M196A-R | ATCTTGAGcgcGTACTCCAGTACTTTACCGCCGA | | |
| HmFLS1-M220A-F | TCACACCgacgcgTCTACCCTCACTATTCTTGTCCCAA | pET28a(+) | SacI/NotI |
| HmFLS1-M220A-R | GTAGAcgcgtcGGTGTGAGCTGGCACCCCAAGT | | |
| HmFLS2-F130A-F | CTATTTCgcgCACGTCATGTCTCCTCCATCCA | pET28a(+) | SacI/NotI |
| HmFLS2-F130A-R | TGACGTGcgcGAAATAGTCAATCCACTCCACCTTC | | |
| HmFLS2-E196A-F | GACTCGAATTTgcgATGAAGATCAACATGTACCCCCC | pET28a(+) | SacI/NotI |
| HmFLS2-E196A-R | CATcgcAAATTCGAGTCCGTCGCCGCCCAGCT | | |
| HmFLS2-M220A-F | ACACCgacgcgtCGGCCCTCACTATACTCGTCC | pET28a(+) | SacI/NotI |
| HmFLS2-M220A-R | GGCCGacgcgtcGGTGTGGGGCTCAACTCCGA | | |

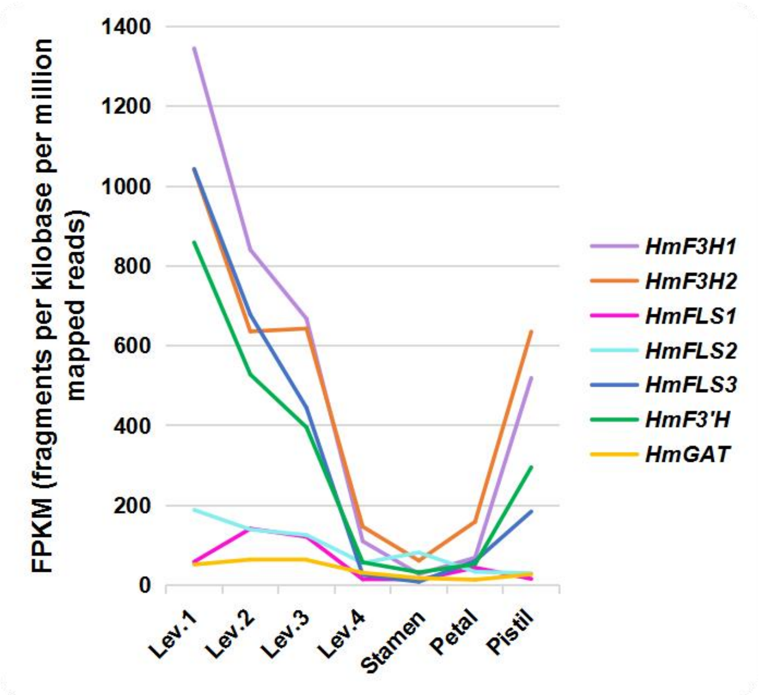


**Figure S1.** FPKM levels of hyperoside-biosynthetic relevant enzymes at different bud stages and tissues of Lev.4 flower buds from *H. monogynum*.

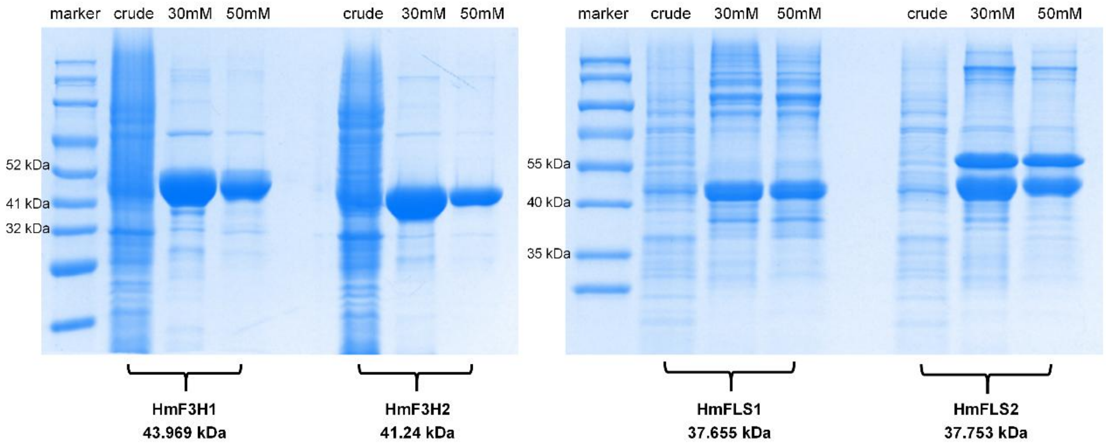


**Figure S2.** SDS-PAGE of $Ni^{2+}$ resin-purified proteins. Crude: crude proteins before imidazole elution; 30mM, 50mM: different concentrations of imidazole elution buffer.

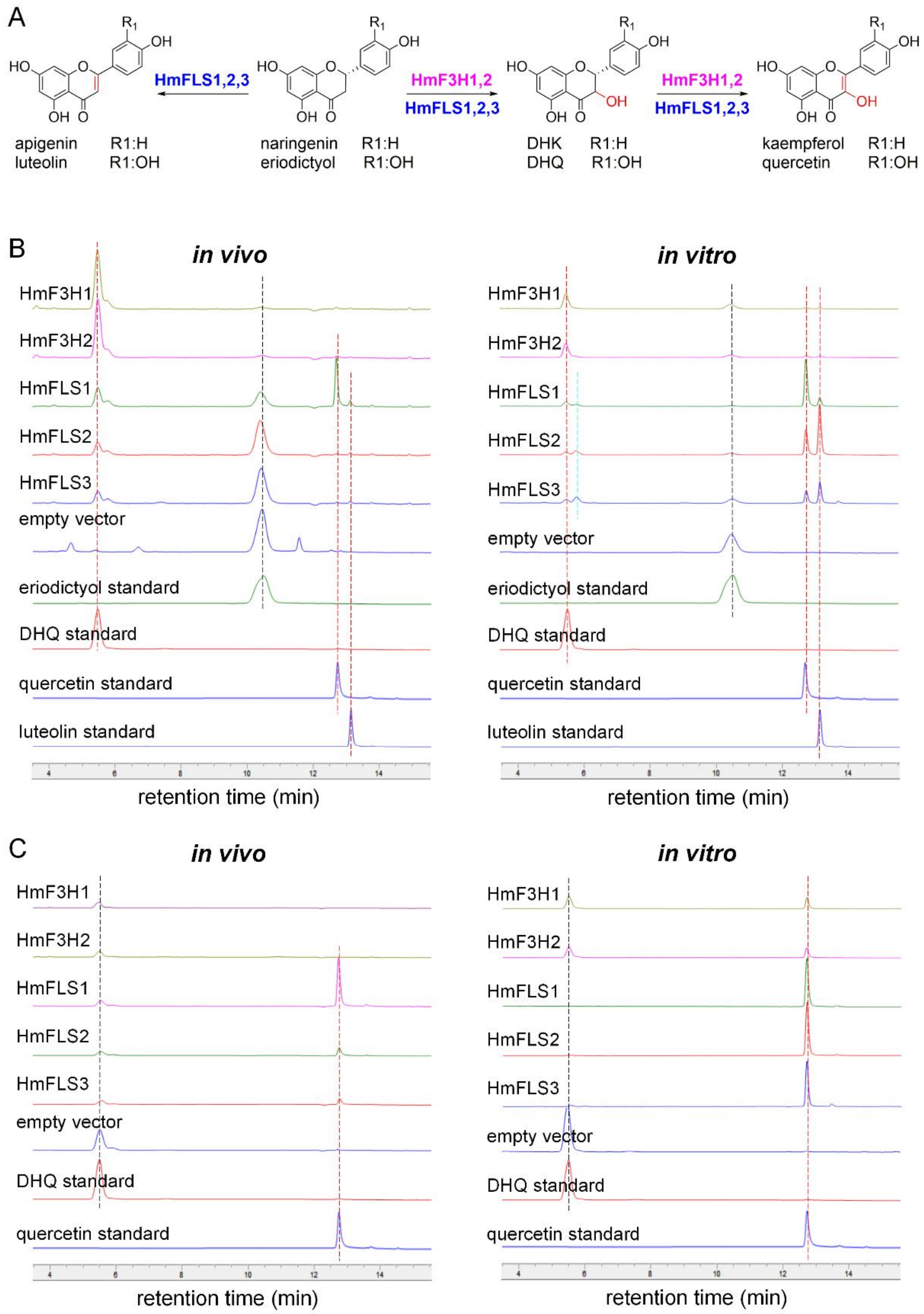


**Figure S3.** *In vivo* and *vitro* activity comparison between HmFLSs and HmF3Hs, with substrates eriodictyol and DHQ. (A) The catalytic scheme of HmF3Hs and HmFLSs. (B) *In vivo* and *vitro* functional discrepancy of HmFLSs and HmF3Hs, with eriodictyol the substrate. (C) *In vivo* and *vitro* functional discrepancy of HmFLSs and HmF3Hs, with DHQ as the substrate. DHQ, dihydroquercetin. Black dashed lines: substrates; red dashed lines: products; turquoise dashed lines: intermediate epimerized product (2R,3S)-*cis*-DHQ. Detection wavelength: 330 nm.

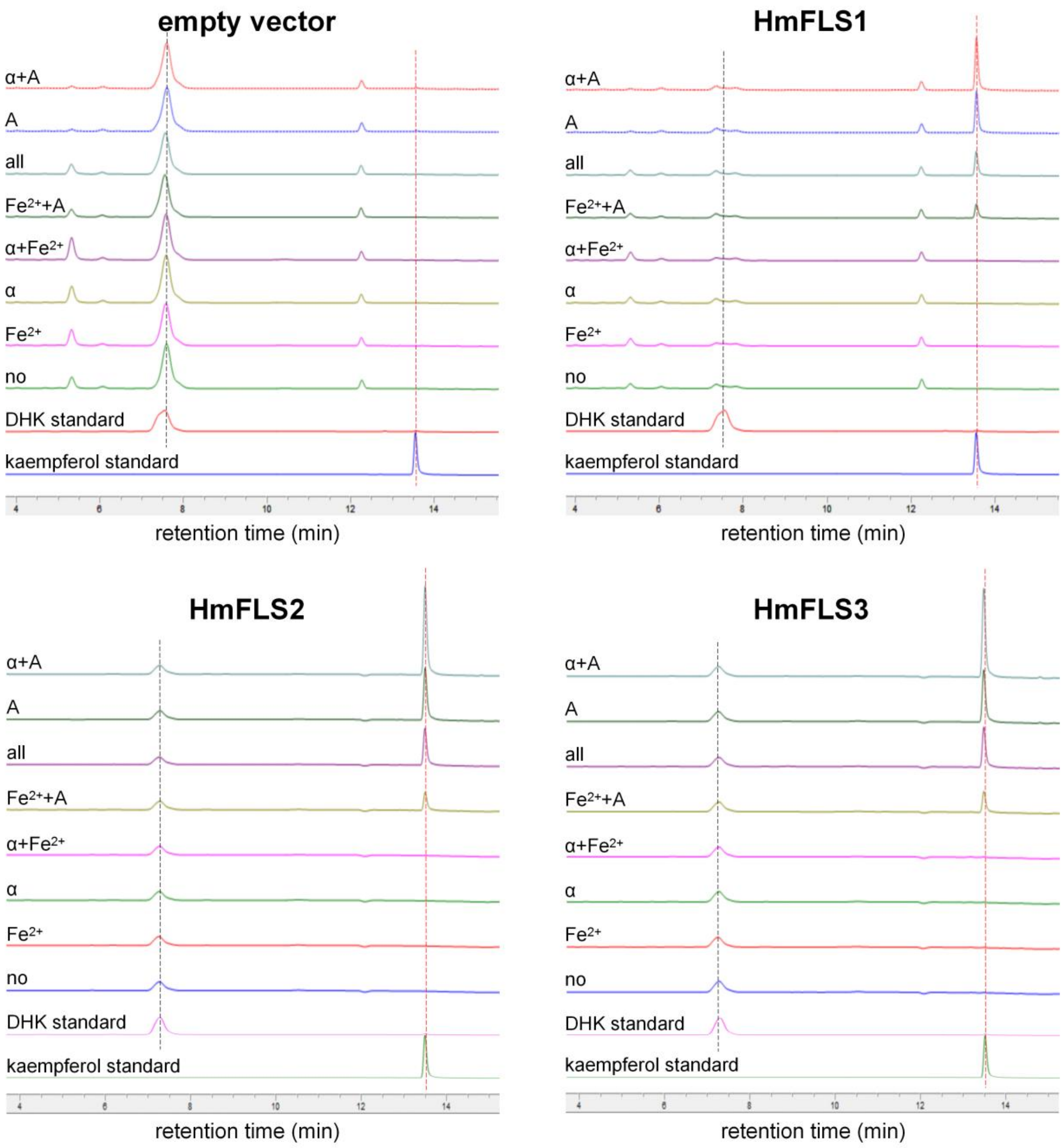


**Figure S4.** The influence of cofactors on HmFLSs catalyzing DHK into kaempferol *in vivo*. Substrates (0.1 mM) and cofactors were added after 5 h IPTG induction in *E. coli* cultures and then incubated for 12 h before ethyl acetate extraction. α, 2.5 mM α-glutaric acid; A, 2.5 mM sodium ascorbate; $Fe^{2+}$: 0.25 mM $FeSO_4$; all, all three cofactors; no, no cofactors; DHK, dihydrokaempferol. Black dashed lines: substrates; red dashed lines: products. Detection wavelength: 360 nm.

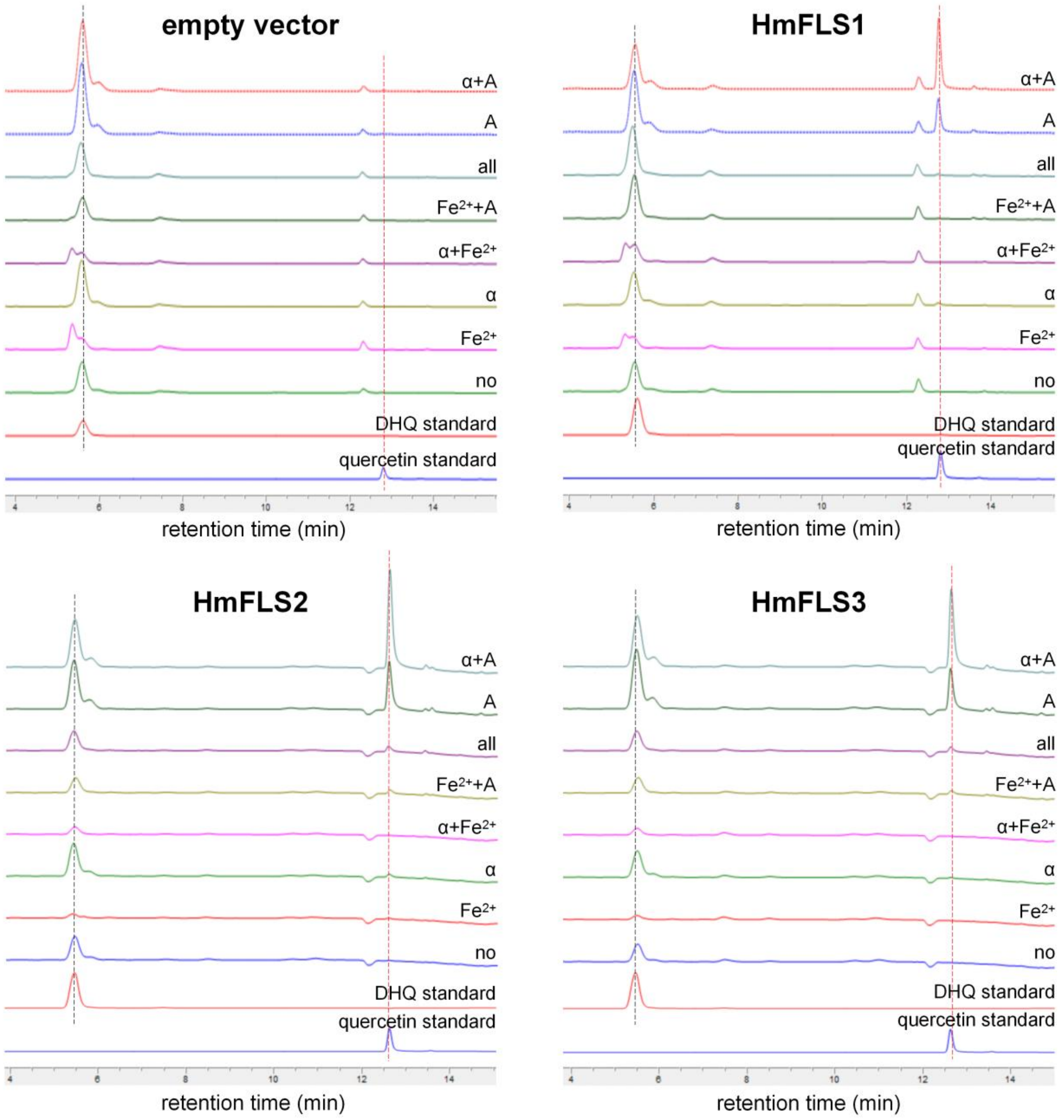


**Figure S5.** The influence of cofactors on HmFLSs catalyzing DHQ into quercetin *in vivo*. Substrates (0.1 mM) and cofactors were added after 5 h IPTG induction in *E. coli* cultures and then incubated for 12 h before ethyl acetate extraction. α, 2.5 mM α-glutaric acid; A, 2.5 mM sodium ascorbate; $Fe^{2+}$: 0.25 mM $FeSO_4$; all, all three cofactors; no, no cofactors; DHQ, dihydroquercetin. Black dashed lines: substrates; red dashed lines: products. Detection wavelength: 360 nm.

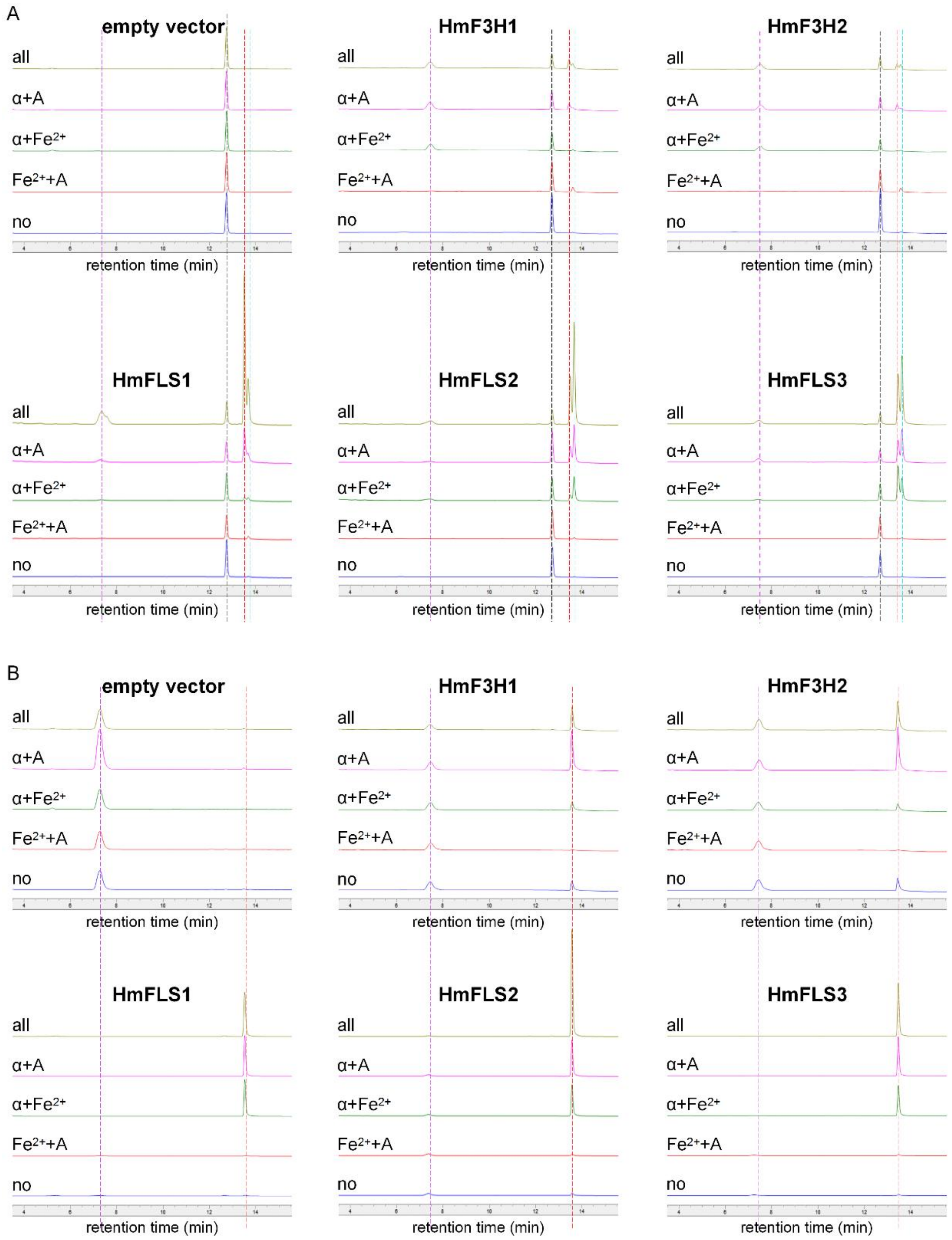


**Figure S6.** The influence of cofactors on HmF3Hs and HmFLSs during *in vitro* verification. (A) The influence of cofactors on the *in vitro* activities of HmF3Hs and HmFLSs, with 0.2 mM naringenin as the substrate. (B) The influence of cofactors on the *in vitro* activities of HmF3Hs and HmFLSs, with 0.2 mM DHK as the substrate. α, 10 mM α-glutaric acid; A, 10 mM sodium ascorbate; $Fe^{2+}$: 0.25 mM $FeSO_4$; all, all three cofactors; no, no cofactors. Black dashed lines: naringenin; purple dashed lines: DHK (dihydrokaempferol); red dashed lines: kaempferol; turquoise dashed lines: apigenin. Detection wavelength: 330 nm.

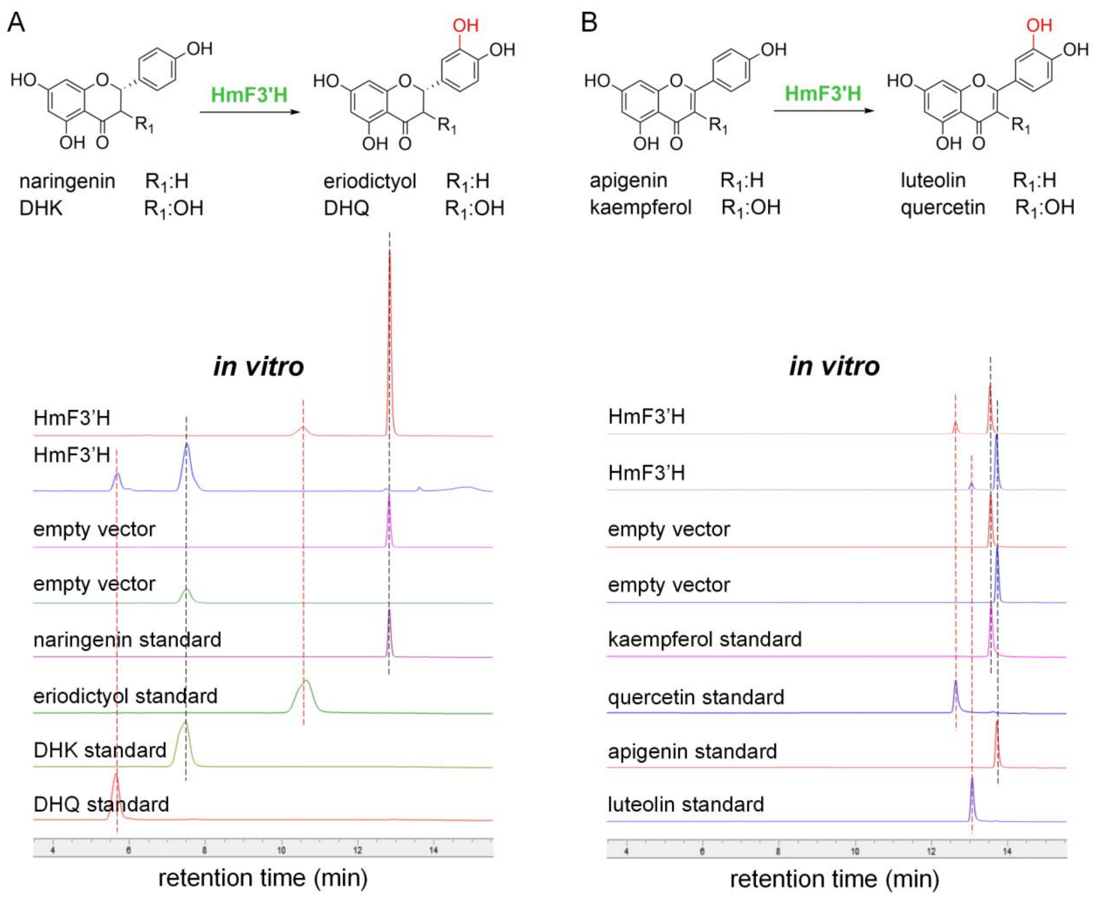


**Figure S7.** *In vitro* verification of HmF3′H with corresponding 3′-hydroxylated products. (A) *In vitro* activity of HmF3′H by catalyzing naringenin and DHK into respective eriodictyol and DHQ. Detection wavelength: 300 nm. (B) *In vitro* activity of HmF3′H by catalyzing kaempferol and apigenin into respective quercetin and luteolin. Detection wavelength: 360 nm. DHK, dihydrokaempferol; DHQ, dihydroquercetin. Black dashed lines: substrates; red dashed lines: products.

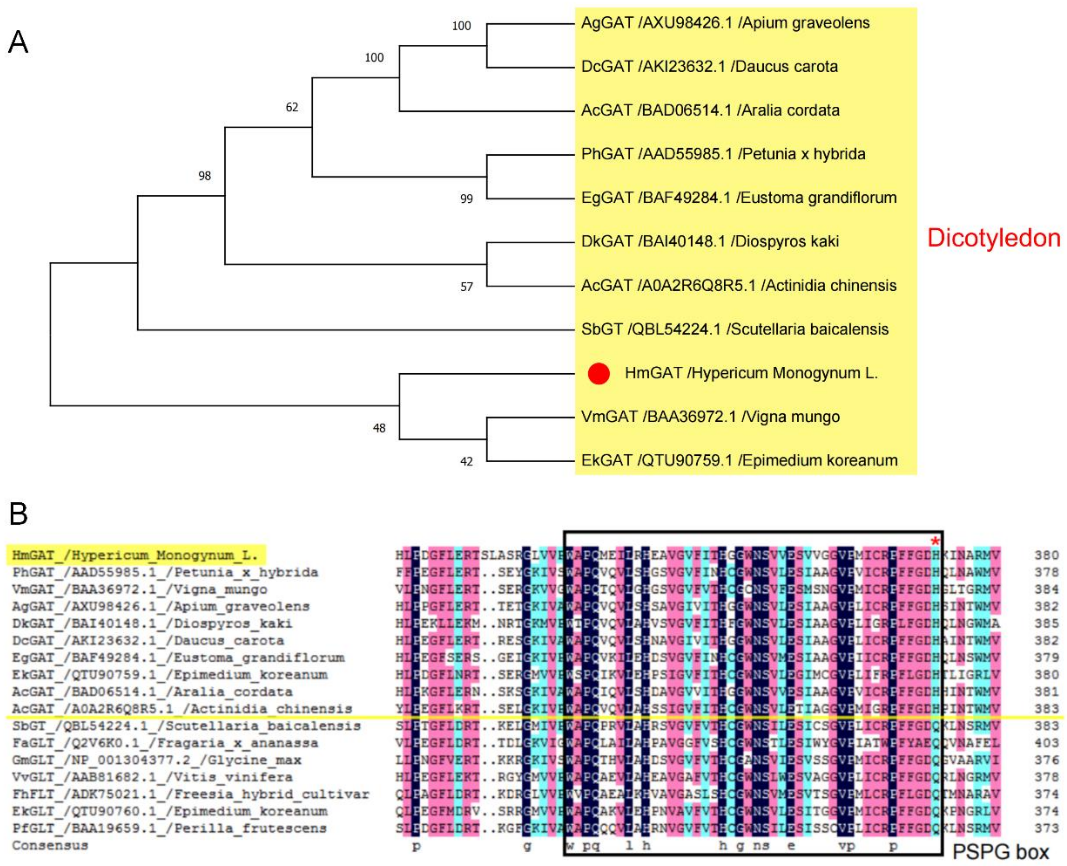


**Figure S8.** The phylogenetic tree and multiple-sequence alignment result of plant GATs. (A) The phylogenetic tree of plant GATs, constructed by MEGA X using Maximum-Likelihood method. The bootstrap value was set as 1000. The numbers on the nodes represent the confidence percentages. (B) The multiple-sequence alignment result of plant GATs and GLTs. The yellow horizontal line separated GATs from GLTs. GAT: flavonoid 3-*O*-galactosyltransferase; GLT: flavonoid 3-*O*-glucosyltransferase. Black box: the conserved PSPG box for glycosyltransferases; Red asterisk: Histidine, the key amino acid residue of plant galactosyltransferases [1].

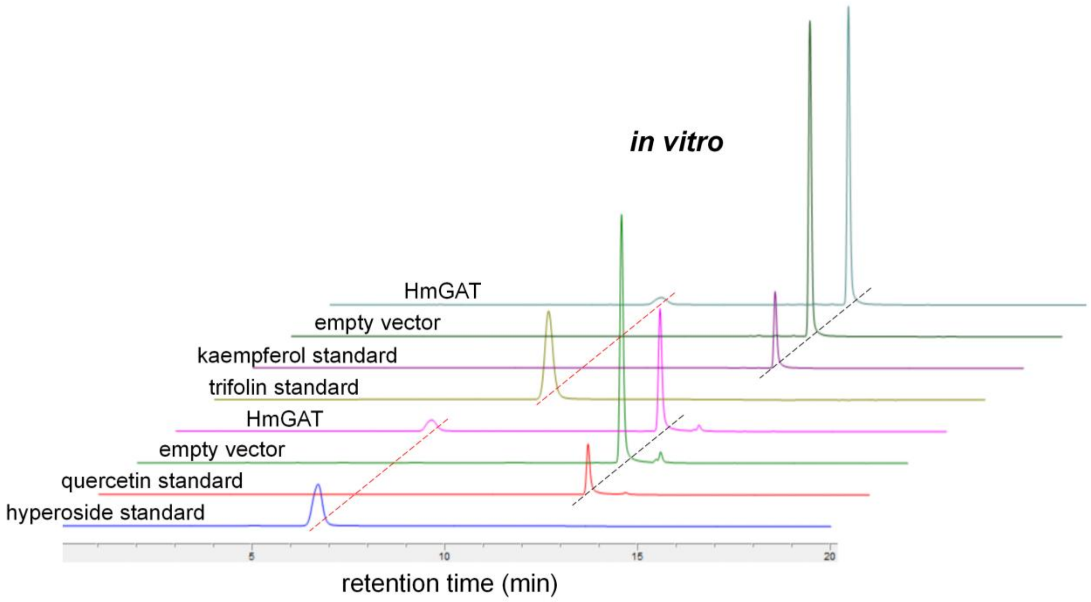


**Figure S9.** HmGAT functional verification *in vitro*, which catalyzed kaempferol and quercetin into trifolin and hyperoside, respectively. Black dashed lines: substrates; red dashed lines: products. Detection wavelength: 360 nm.

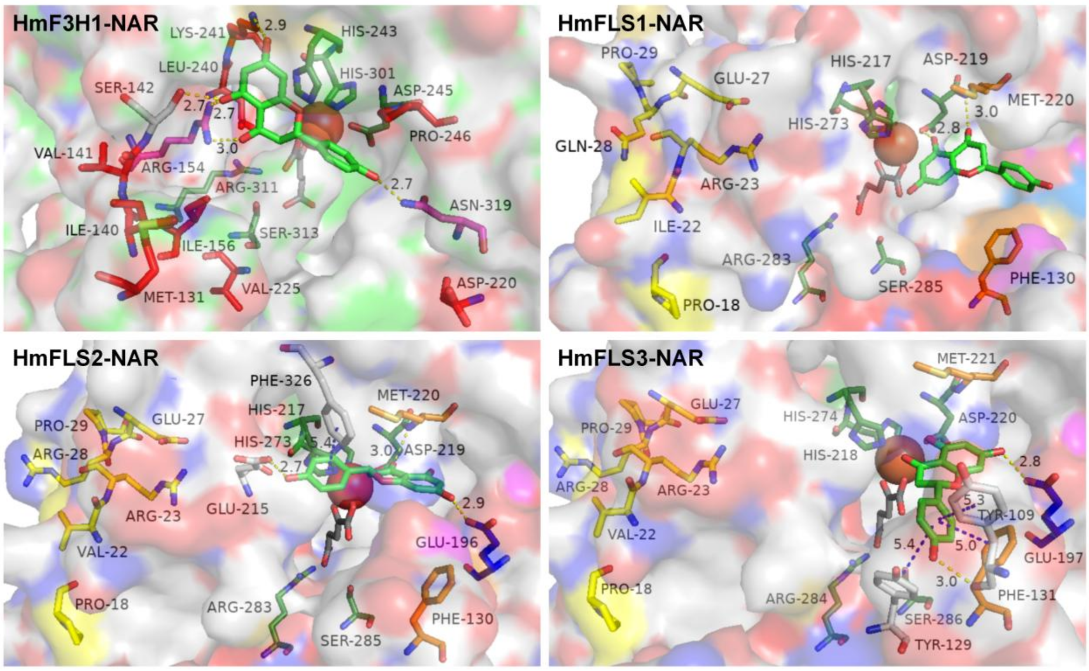


**Figure S10.** The overall molecular docking results of HmF3H1 and HmFLS1-3 with NAR, respectively. NAR, naringenin; dark gray compound, α-glutaric acid. Yellow dashed lines: H-bond interactions. Blue dashed lines: π-π stacking interactions. Dark green residues: conserved $HxDx_nH$ (for $Fe^{2+}$ binding) and RxS (for substrate binding) motifs across 2-ODD enzymes [2]. Red residues: conserved amino acid residues for F3Hs [2-4]. Yellow residues: conserved PxxxIRxxxEQP motif for FLSs [2, 5]. Magenta residues: newly identified conserved amino acid residues for F3Hs. Orange residues: newly identified conserved amino acid residues for FLSs. Blue residue: the conserved amino acid residue for HmFLS2-3.

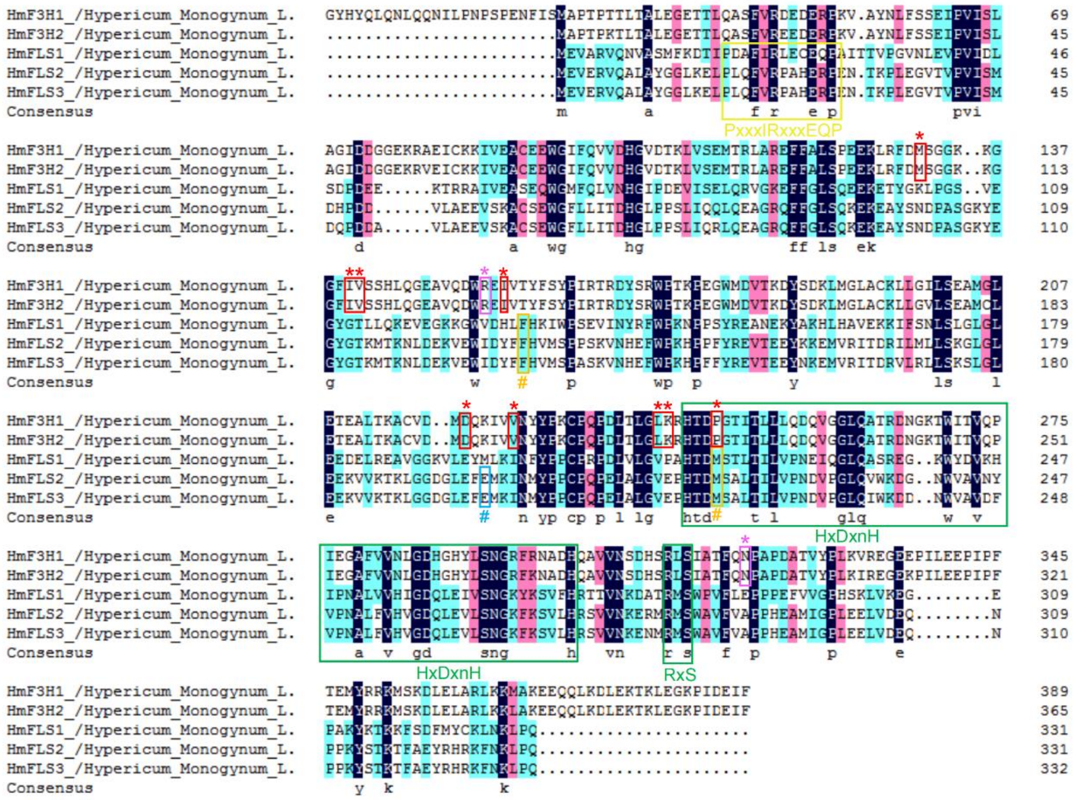


**Figure S11.** Multiple-sequence alignment result of HmF3Hs and HmFLSs. Green boxes: $HxDx_nH$ ($Fe^{2+}$-binding motif) and RxS (substrate-binding motif), conserved across 2-ODDs. Red asterisks: the conserved amino acid residues for F3Hs. Yellow box: PxxxIRxxxEQP motif, conserved for FLSs. Magenta asterisks: the newly identified conserved amino acid residues for F3Hs. Orange pound signs, the newly identified conserved amino acid residues for FLSs. Blue pound sign, the conserved amino acid residue for HmFLS2-3.

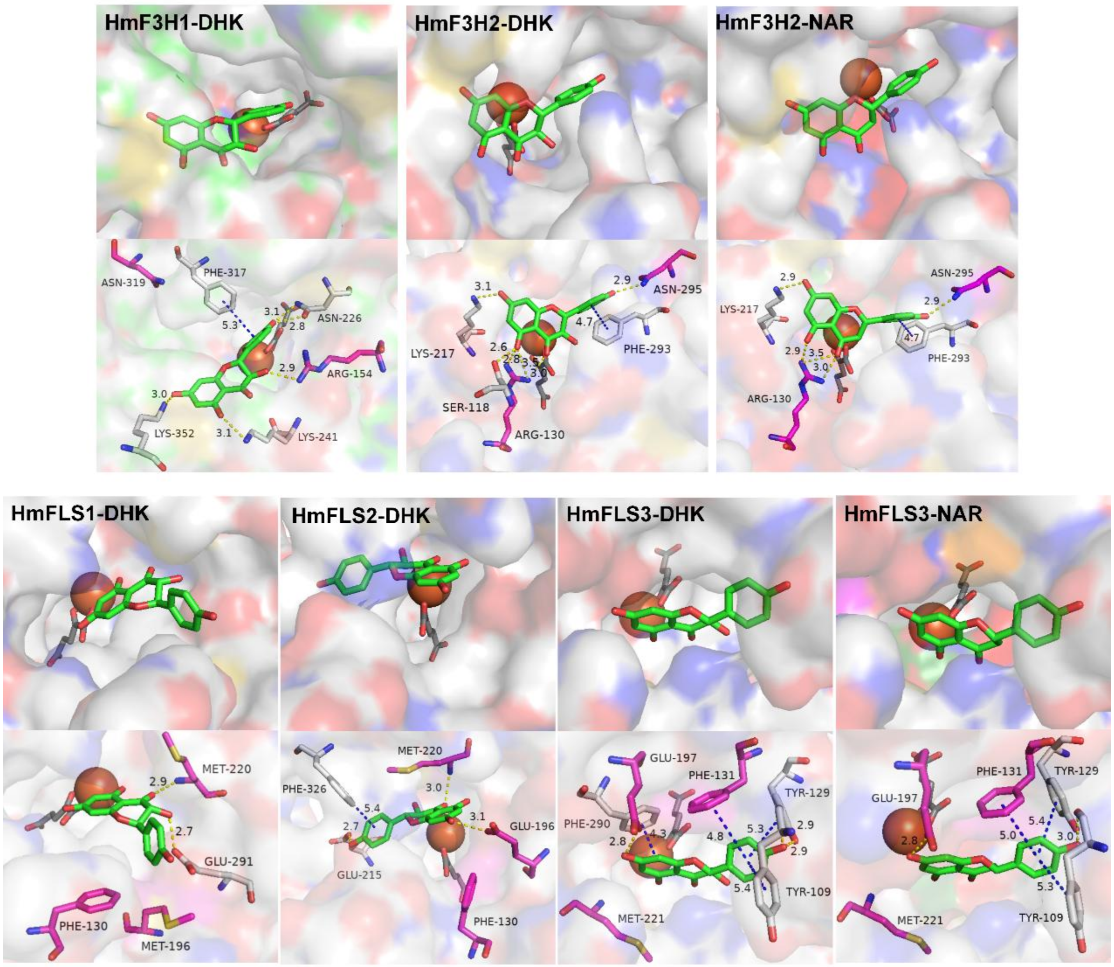


**Figure S12.** Molecular docking results of HmF3Hs and HmFLSs with NAR and DHK, respectively. NAR, naringenin; DHK, dihydrokaempferol; dark gray compound, α-glutaric acid. Yellow dashed lines: H-bond interactions. Blue dashed lines: π-π stacking interactions. Magenta residues: newly identified conserved amino acid residues.

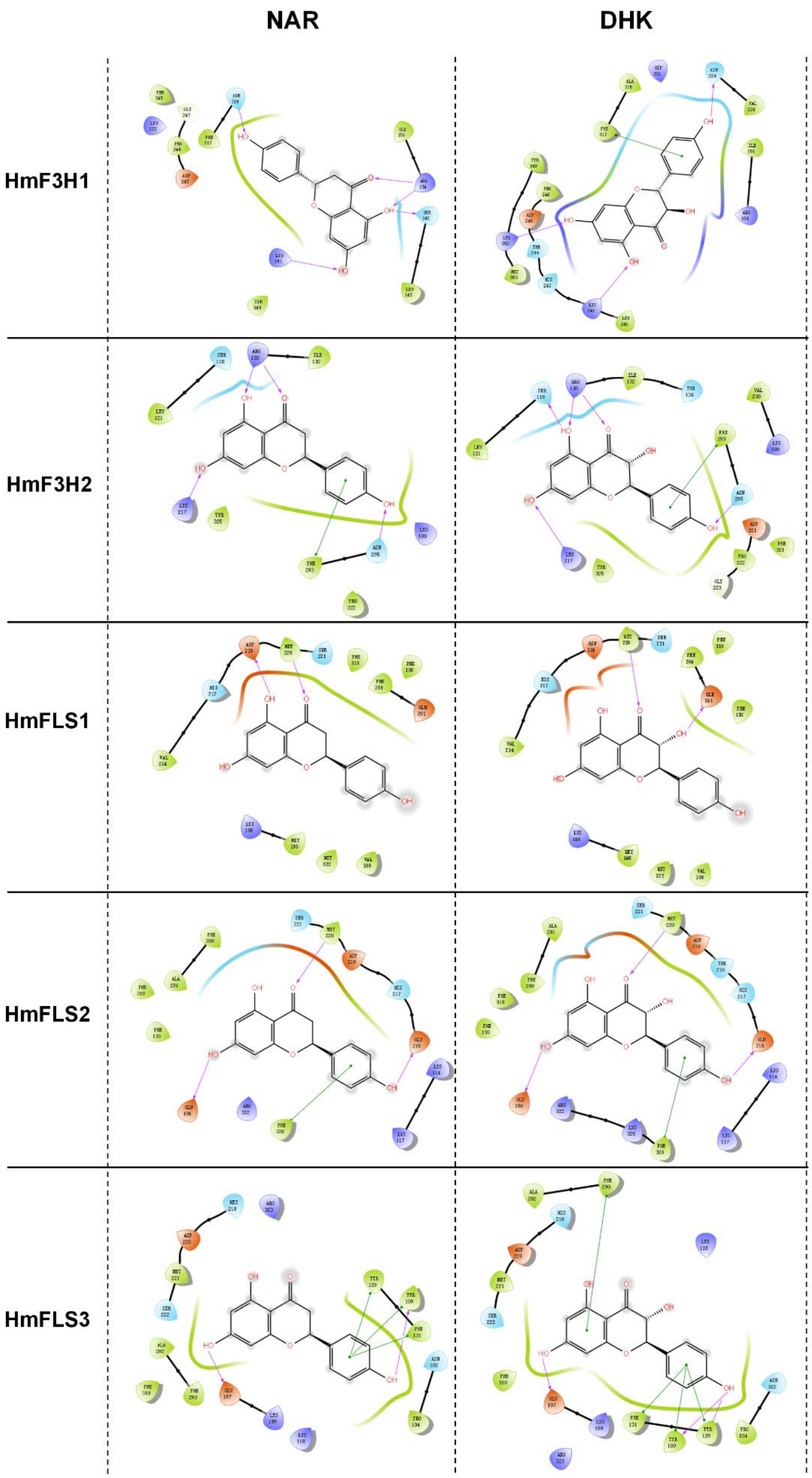


**Figure S13.** The overall molecular docking results of HmF3Hs and HmFLSs with two substrate ligands (NAR, DHK) displayed in diagrams. NAR, naringenin; DHK, dihydrokaempferol. Purple arrows: H-bond interactions. Green lines: π-π stacking interactions.

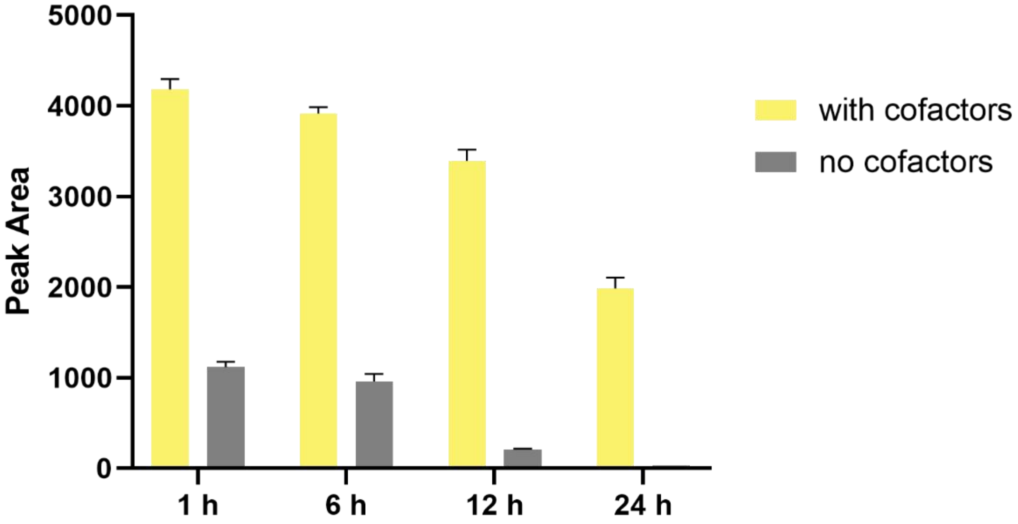


**Figure S14.** Hyperoside production in *E. coli* BL21(DE3) strain catalyzed by HmGAT with/without cofactors at different time points. The recombinant strain was added with 0.1 mM quercetin plus cofactors (2.5 mM ascorbate + 2.5 mM α-glutaric acid) after 5 h IPTG induction. Values represented the means of three independent experiments ± standard deviation.

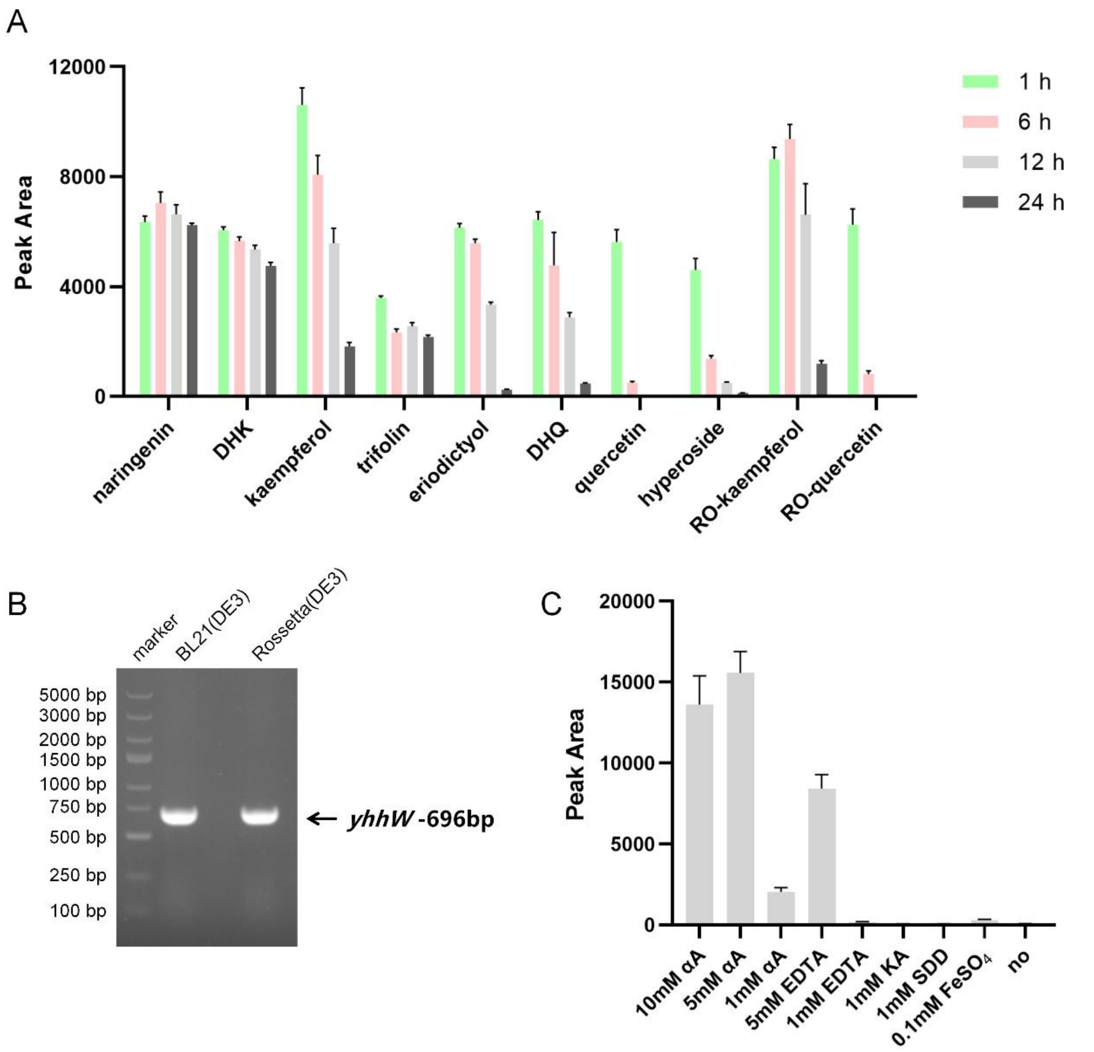


**Figure S15.** Flavonoids decomposition in *E. coli* mediated by yhhW. (A) The contents of distinct flavonoids in *E. coli* BL21(DE3) and Rossetta(DE3) strains at different time points. DHK, dihydrokaempferol; DHQ, dihydroquercetin; RO, Rossetta(DE3) strain. Strains were added with 0.1 mM flavonoids after 5 h IPTG induction. (B) PCR cloning result of *yhhW* gene (696 bp) in *E. coli* BL21(DE3) *and* Rossetta(DE3). (C) The effect of different cofactors on quercetin decomposition in *E. coli* BL21(DE3). *E. coli* BL21(DE3) was added with 0.2 mM quercetin plus different cofactors after 7 h IPTG induction, then incubated for 15 h before ethyl acetate extraction. A, ascorbic acid; α, α-glutaric acid; EDTA, ethylene diamine tetra-acetic acid disodium salt; KA, kojic acid; SDD, sodium diethyldithiocarbamate; no, no cofactors. Values represented the means of three independent experiments ± standard deviation.

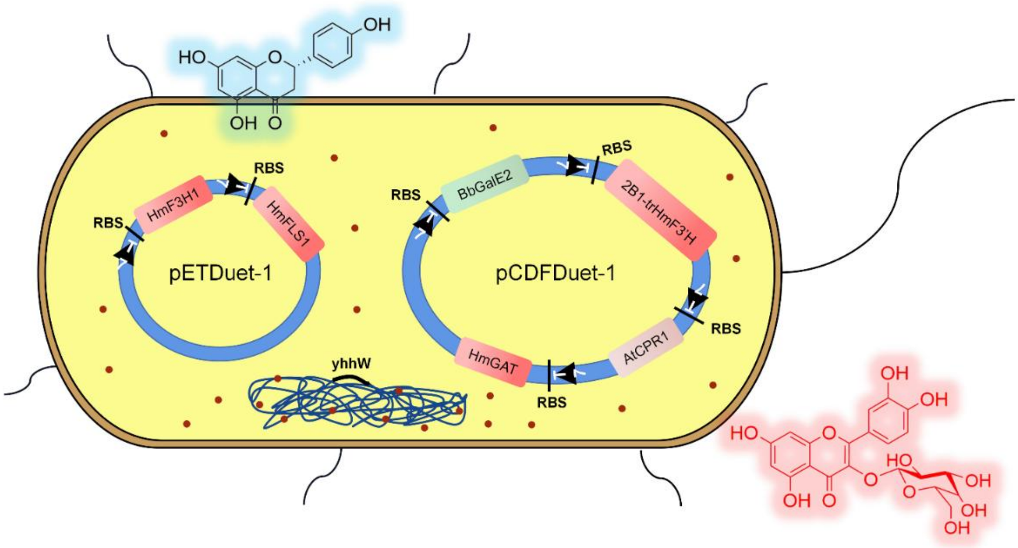


**Figure S16.** The layout of engineered *E. coli* BL21(DE3) factory (type I-b) for producing hyperoside. trHmF3′H: N-terminal transmembrane signal peptide-truncated HmF3′H; BbGalE2: *B. bifidum* UDP-glucose 4-epimerase 2; AtCPR1: *A. thaliana* CYP450 reductase 1.

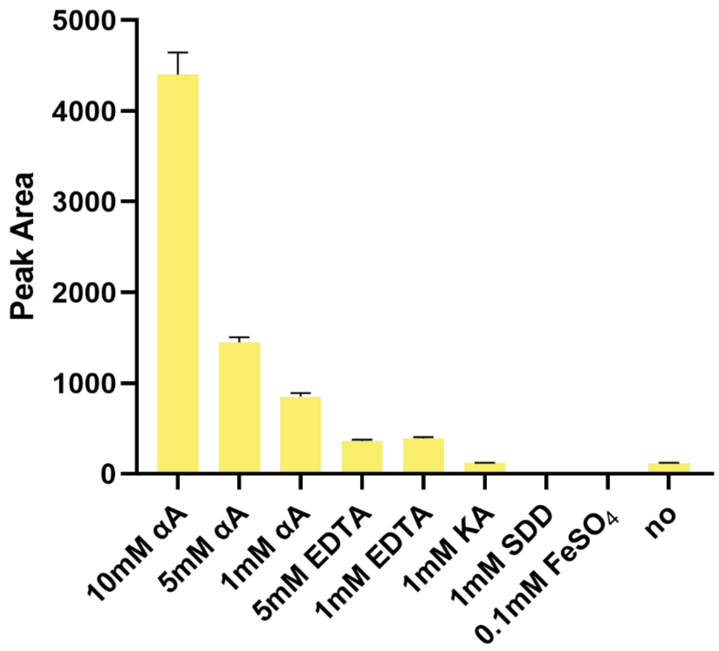


**Figure S17.** Hyperoside production in type I-b *E. coli* BL21(DE3) strain under different cofactors. Type I-b strain was fed with 0.3 mM naringenin plus different cofactors for 15 h after 7 h IPTG (1 mM) induction. A, ascorbic acid; α, α-glutaric acid; EDTA, ethylene diamine tetra-acetic acid disodium salt; KA, kojic acid; SDD, sodium diethyldithiocarbamate; no, no cofactors. Values represented the means of three independent experiments ± standard deviation.

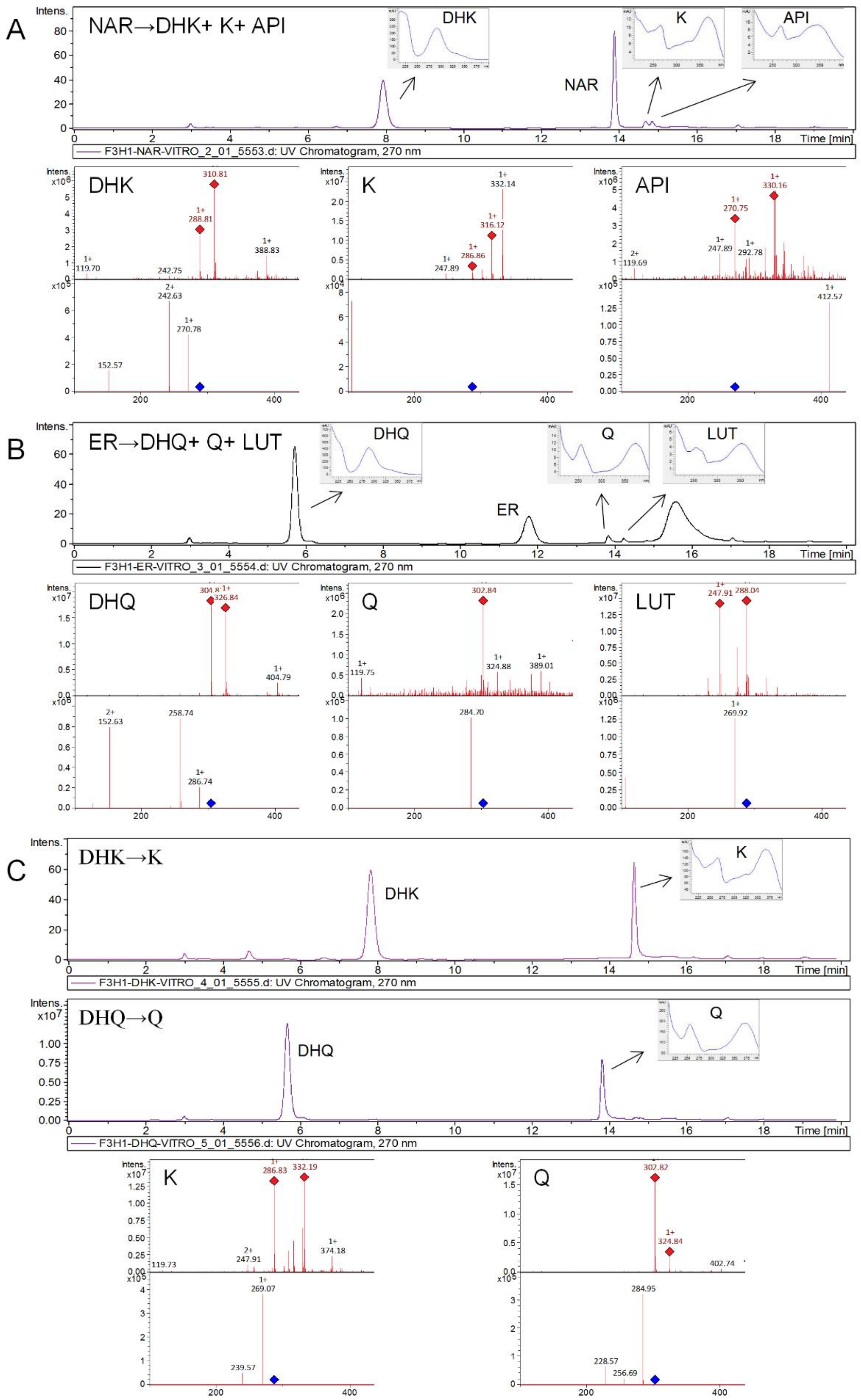


**Figure S18.** The LC-MS/MS spectra and UV chromatograms of *in vitro* catalytic products by HmF3H1 with four substrates. (A) NAR as the substrate to produce DHK, K, and API. (B) ER as the substrate to produce DHQ, Q, and LUT. (C) DHK and DHQ as respective substrate to produce K and Q. NAR, naringenin; ER, eriodictyol; DHK, dihydrokaempferol; DHQ, dihydroquercetin; K, kaempferol; Q, quercetin; API, apigenin; LUT, luteolin. Detection wavelength: 270 nm.

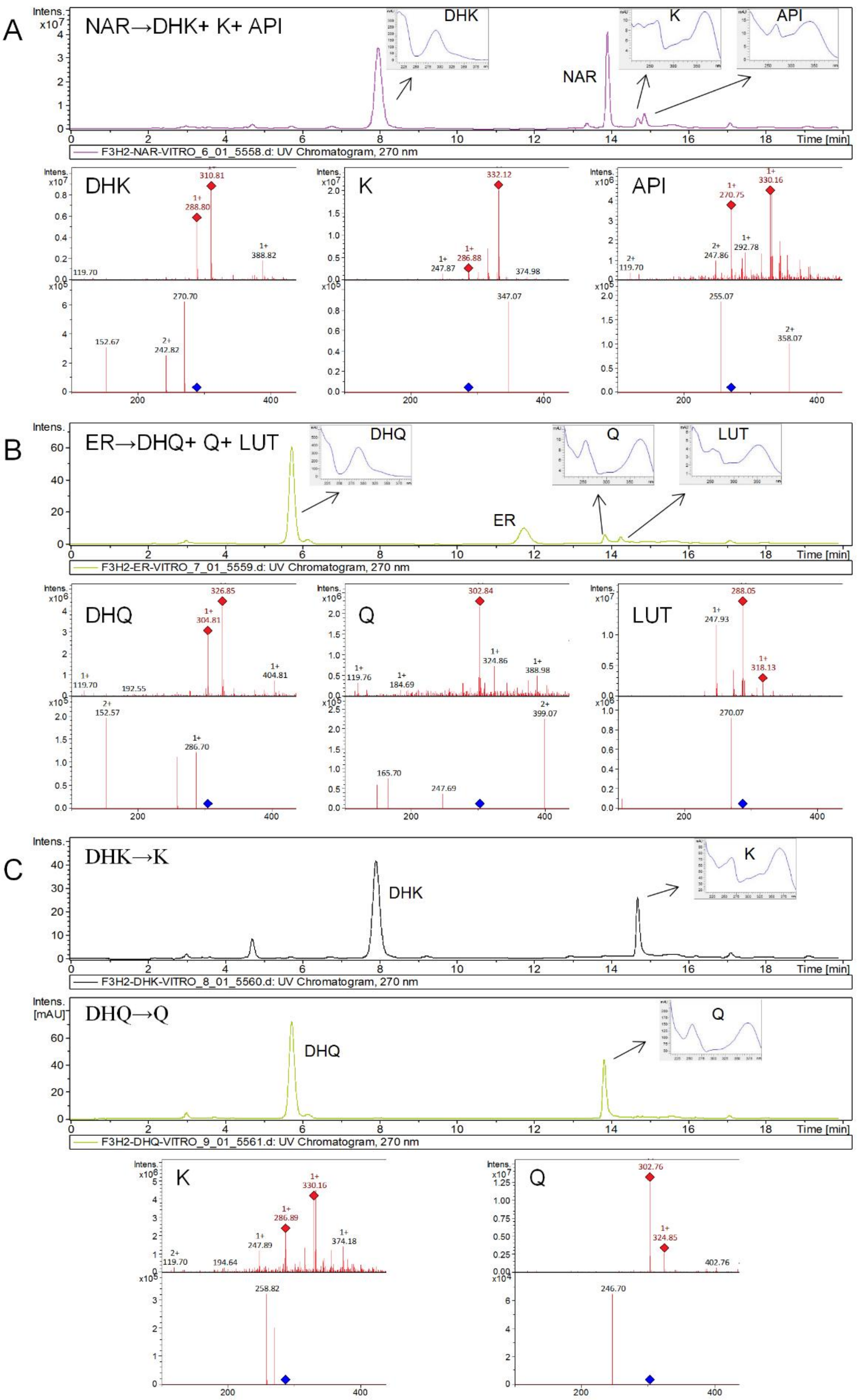


**Figure S19.** The LC-MS/MS spectra and UV chromatograms of *in vitro* catalytic products by HmF3H2 with four substrates. (A) NAR as the substrate to produce DHK, K, and API. (B) ER as the substrate to produce DHQ, Q, and LUT. (C) DHK and DHQ as respective substrate to produce K and Q. Detection wavelength: 270 nm.

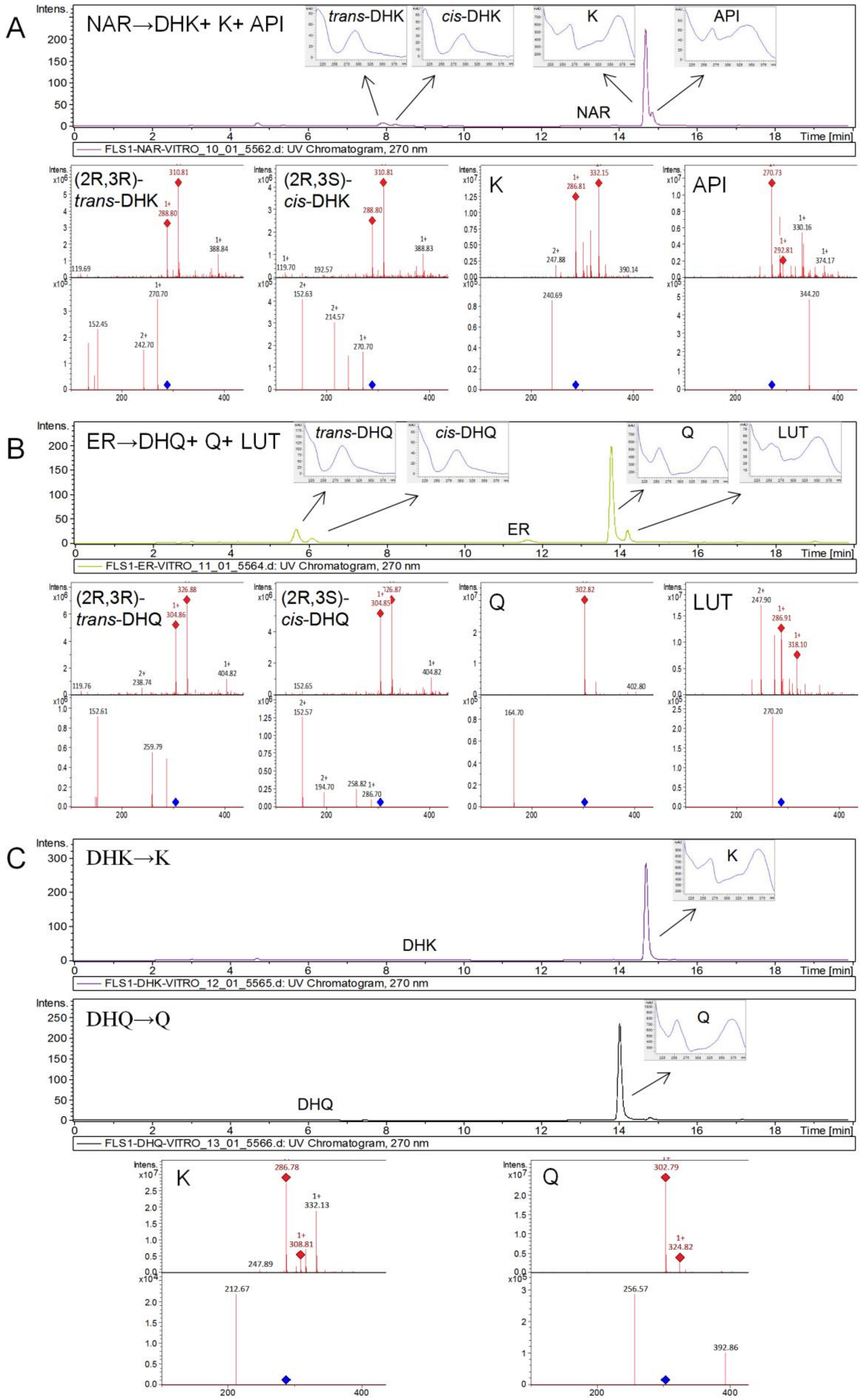


**Figure S20.** The LC-MS/MS spectra and UV chromatograms of *in vitro* catalytic products by HmFLS1 with four substrates. (A) NAR as the substrate to produce DHK, K, and API. (B) ER as the substrate to produce DHQ, Q, and LUT. (C) DHK and DHQ as respective substrate to produce K and Q. Detection wavelength: 270 nm.

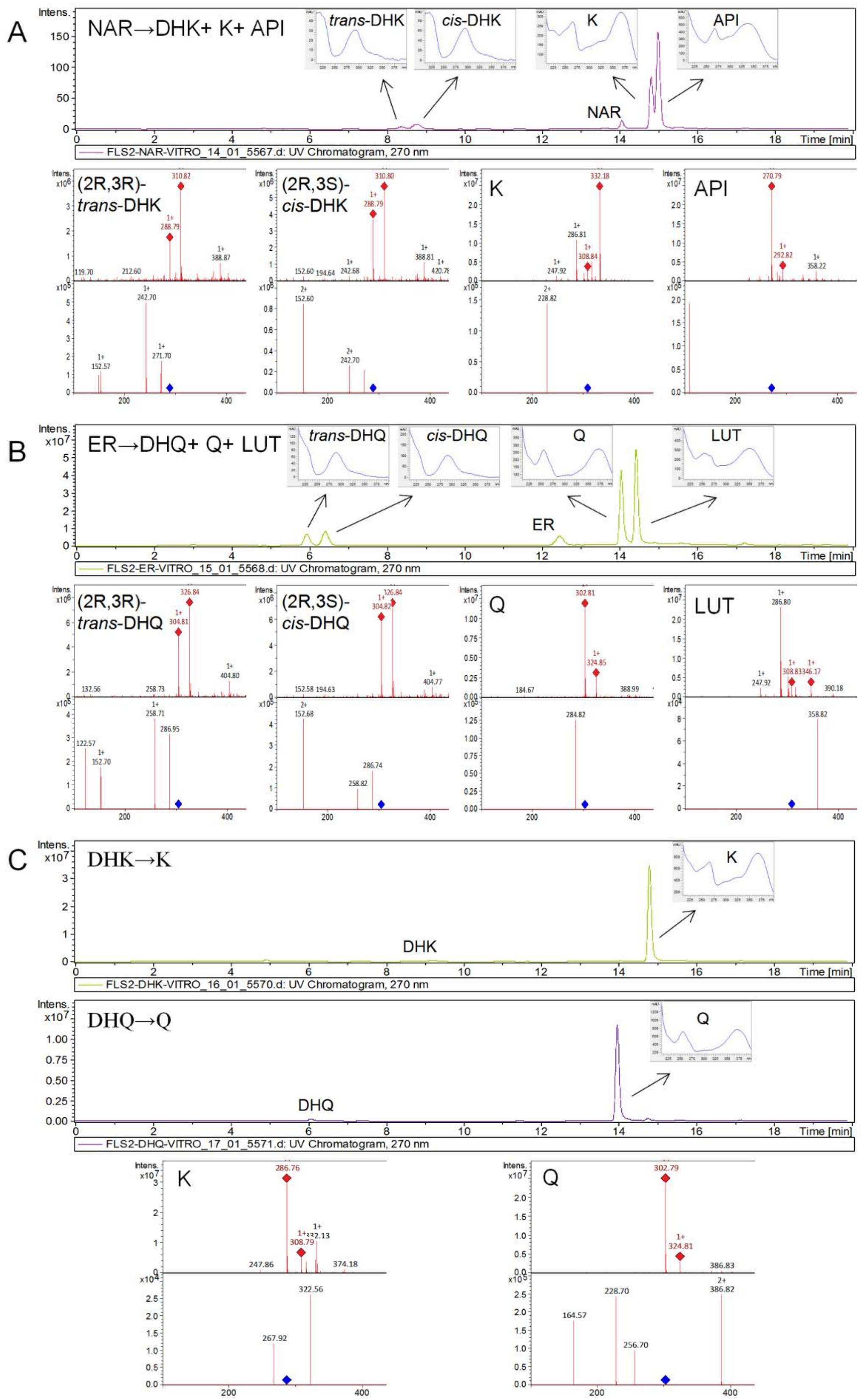


**Figure S21.** The LC-MS/MS spectra and UV chromatograms of *in vitro* catalytic products by HmFLS2 with four substrates. (A) NAR as the substrate to produce DHK, K, and API. (B) ER as the substrate to produce DHQ, Q, and LUT. (C) DHK and DHQ as respective substrate to produce K and Q. Detection wavelength: 270 nm.

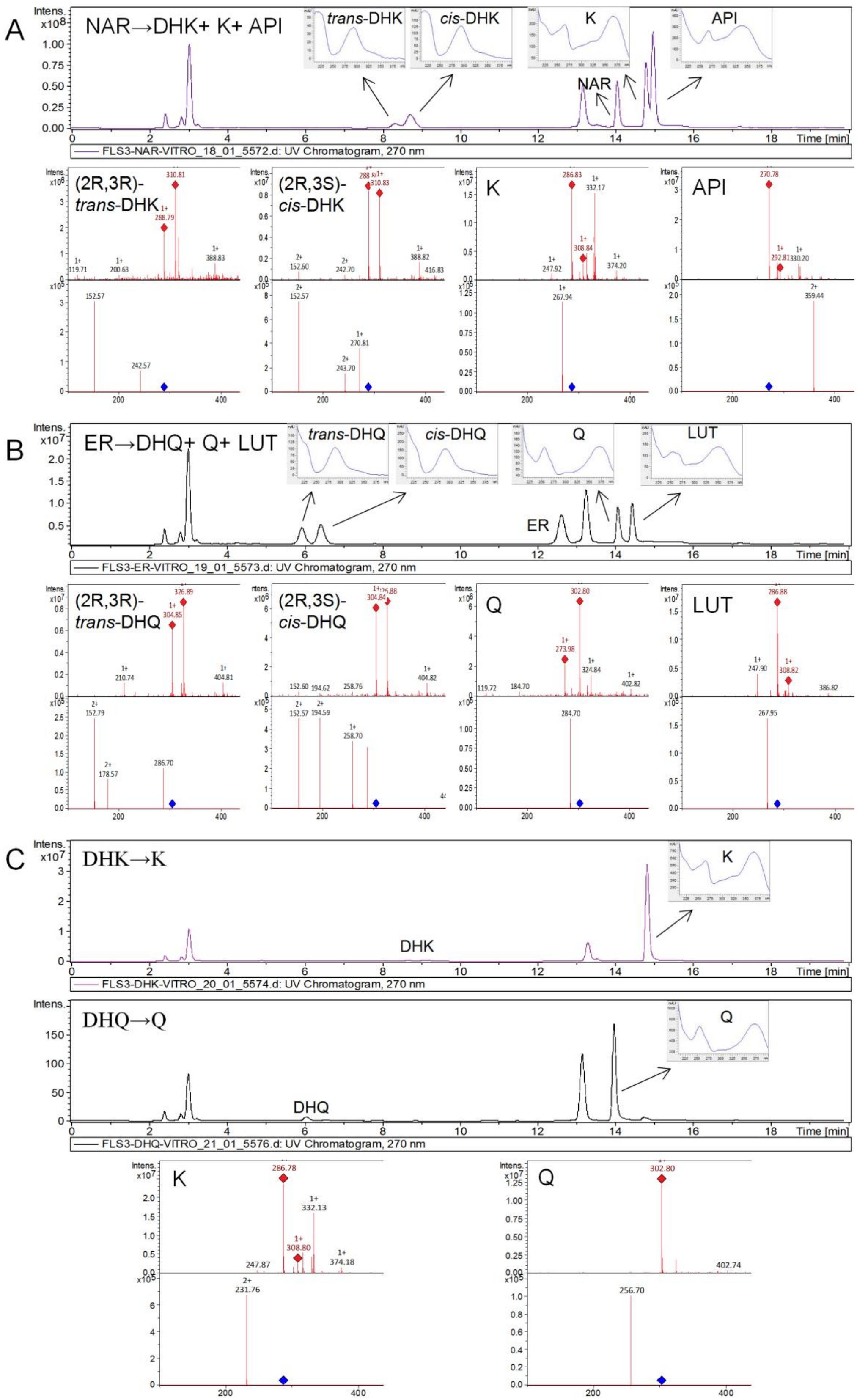


**Figure S22.** The LC-MS/MS spectra and UV chromatograms of *in vitro* catalytic products by HmFLS3 with four substrates. (A) NAR as the substrate to produce DHK, K, and API. (B) ER as the substrate to produce DHQ, Q, and LUT. (C) DHK and DHQ as respective substrate to produce K and Q. Detection wavelength: 270 nm.

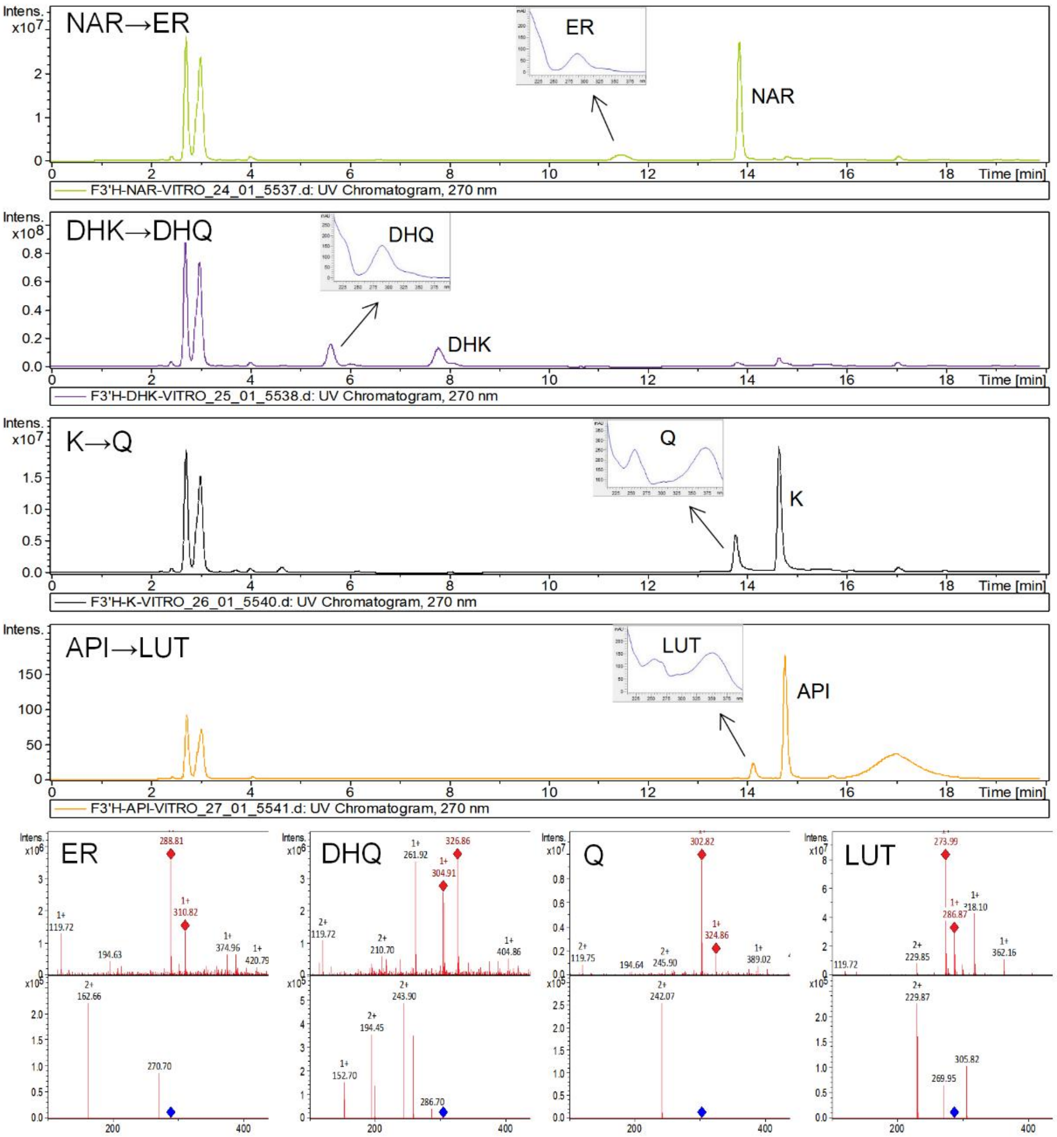


**Figure S23.** The LC-MS/MS spectra and UV chromatograms of *in vitro* catalytic products by HmF3′H, with NAR, DHK, K, and API as respective substrate. Detection wavelength: 270 nm.

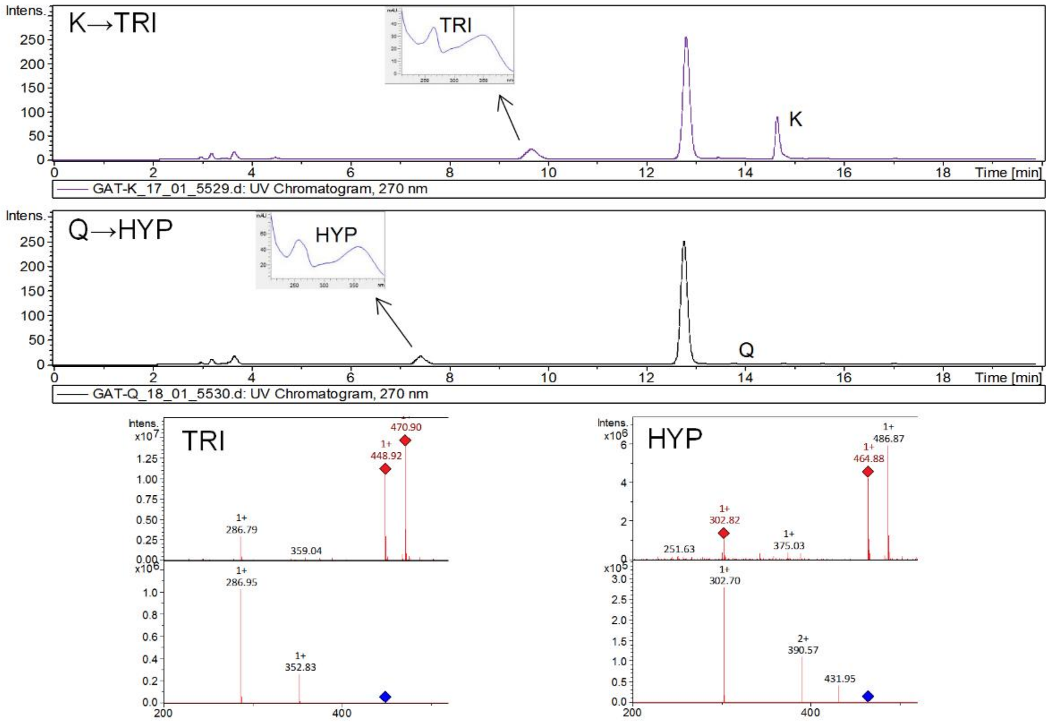


**Figure S24.** The LC-MS/MS spectra and UV chromatograms of *in vitro* catalytic products by HmGAT, with K and Q as respective substrate. TRI, trifolin; HYP, hyperoside. Detection wavelength: 270 nm.

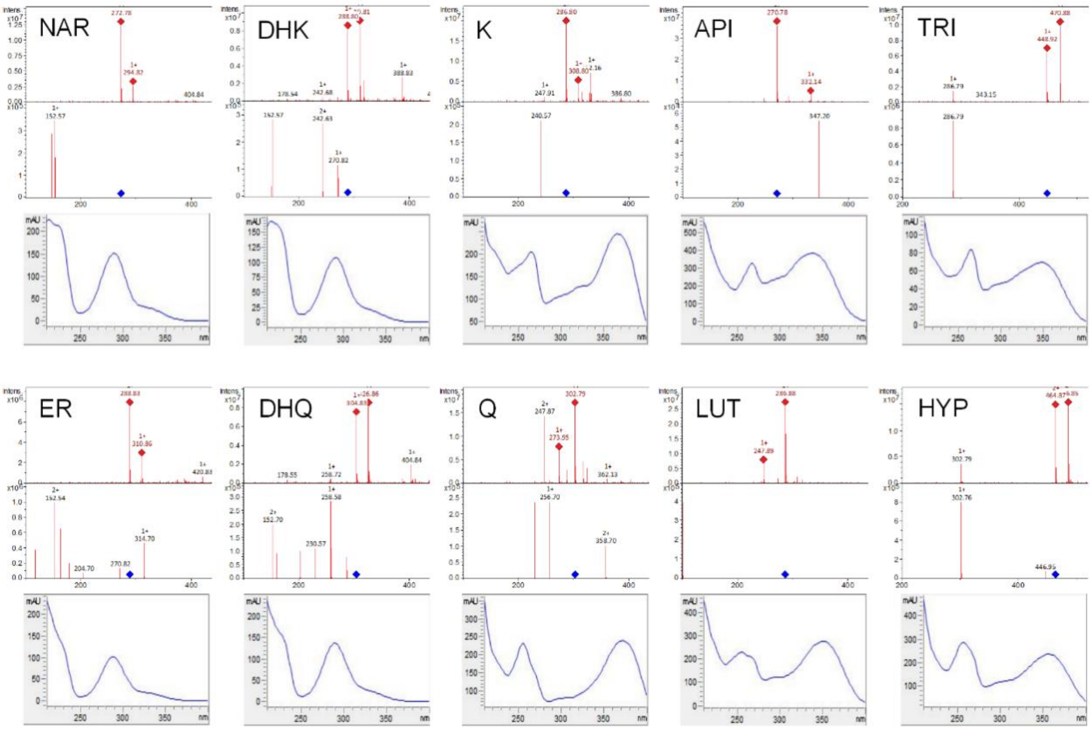


**Figure S25.** The LC-MS/MS spectra and UV chromatograms of the compound standards. NAR, naringenin; ER, eriodictyol; DHK, dihydrokaempferol; DHQ, dihydroquercetin; K, kaempferol; Q, quercetin; API, apigenin; LUT, luteolin; TRI, trifolin; HYP, hyperoside. Detection wavelength: 270 nm.

## References


1. Ikegami A, Akagi T, Potter D et al. Molecular identification of 1-cys peroxiredoxin and anthocyanidin/flavonol 3-o-galactosyltransferase from proanthocyanidin-rich young fruits of persimmon (diospyros kaki thunb.). *Planta*. 2009;**230**:841-855.
2. Cheng AX, Han XJ, Wu YF et al. The function and catalysis of 2-oxoglutarate-dependent oxygenases involved in plant flavonoid biosynthesis. *Int. J. Mol. Sci.* 2014;**15**:1080-1095.
3. Gebhardt YH, Witte S, Steuber H et al. Evolution of flavone synthase i from parsley flavanone 3beta-hydroxylase by site-directed mutagenesis. *Plant Physiol.* 2007;**144**:1442-1454.
4. Li DD, Ni R, Wang PP et al. Molecular basis for chemical evolution of flavones to flavonols and anthocyanins in land plants. *Plant Physiol.* 2020;**184**:1731-1743.
5. Owens DK, Alerding AB, Crosby KC et al. Functional analysis of a predicted flavonol synthase gene family in arabidopsis. *Plant Physiol.* 2008;**147**:1046-1061.
